# Stability, change, and reliable individual differences in electroencephalography measures: A lifespan perspective on progress and opportunities

**DOI:** 10.1016/j.neuroimage.2023.120116

**Published:** 2023-07-15

**Authors:** K.L. Lopez, A.D. Monachino, K.M. Vincent, F.C. Peck, L.J. Gabard-Durnam

**Affiliations:** a Northeastern University, 360 Huntington Ave, Boston, MA, United States; b University of California, Los Angeles, Los Angeles, CA, United States

**Keywords:** EEG, Individual differences, Psychometric reliability, Internal consistency, Test-retest reliability, Lifespan

## Abstract

Electroencephalographic (EEG) methods have great potential to serve both basic and clinical science approaches to understand individual differences in human neural function. Importantly, the psychometric properties of EEG data, such as internal consistency and test-retest reliability, constrain their ability to differentiate individuals successfully. Rapid and recent technological and computational advancements in EEG research make it timely to revisit the topic of psychometric reliability in the context of individual difference analyses. Moreover, pediatric and clinical samples provide some of the most salient and urgent opportunities to apply individual difference approaches, but the changes these populations experience over time also provide unique challenges from a psychometric perspective. Here we take a developmental neuroscience perspective to consider progress and new opportunities for parsing the reliability and stability of individual differences in EEG measurements across the lifespan. We first conceptually map the different profiles of measurement reliability expected for different types of individual difference analyses over the lifespan. Next, we summarize and evaluate the state of the field's empirical knowledge and need for testing measurement reliability, both internal consistency and test-retest reliability, across EEG measures of power, event-related potentials, nonlinearity, and functional connectivity across ages. Finally, we highlight how standardized pre-processing software for EEG denoising and empirical metrics of individual data quality may be used to further improve EEG-based individual differences research moving forward. We also include recommendations and resources throughout that individual researchers can implement to improve the utility and reproducibility of individual differences analyses with EEG across the lifespan.

## Highlights

• We detail EEG psychometric reliability profiles for testing individual differences.
• We review internal consistency of power, ERP, nonlinear, connectivity measures.
• We review test-retest reliability of power, ERP, nonlinear, connectivity measures.
• We show how denoising, data quality measures improve individual difference studies.
• We provide actionable recommendations to improve EEG individual difference analyses.

## 1 Introduction

There is great momentum within both basic and clinical human science to apply individual difference approaches to brain research. Examining differences between individuals is an important means to understand how variation in environmental experiences impacts the brain, and how brain measures relate to other systems and levels of measurement, like differences in cognition or behavior (e.g. Ambrosini and Vallesi, 2016; Drew and Vogel, 2008; Hakim et al., 2021; Hodel et al., 2019; Jones et al., 2020; Sanchez-Alonso and Aslin, 2020; Vogel et al., 2005; Vogel and Machizawa, 2004). These approaches are also critical for efforts towards precision clinical science, including identification of brain-based biomarkers for risk, health status, outcomes, intervention targets, and treatment response indicators (e.g. Bosl et al., 2018; de Aguiar Neto and Rosa, 2019; Frohlich et al., 2019; Furman et al., 2018; Gabard-Durnam et al., 2019; Geuter et al., 2018; Hannesdóttir et al., 2010; Jones et al., 2020; Moser et al., 2015; Stewart et al., 2011; Wilkinson et al., 2020). Developmental populations provide additional opportunities to apply individual difference approaches, including linking early brain measure differences to subsequent emergent behaviors or symptoms and mapping heterogeneity in brain development trajectories in both basic and clinical contexts (e.g. Bosl et al., 2018; Frohlich et al., 2019; Gabard-Durnam et al., 2019; Hannesdóttir et al., 2010; Hodel et al., 2019; Jones et al., 2020; Moser et al., 2015; Sanchez-Alonso and Aslin, 2020; Wilkinson et al., 2020).

Importantly, the validity of brain measurements to serve these purposes in individual differences research is constrained by their psychometric reliability (Parsons et al., 2019). That is, if there is insufficient certainty about individuals’ brain estimates due to error, it will be impossible to derive meaningful associations between those brain estimates and individual phenotypes. There are multiple approaches to conceptualizing, measuring, and interpreting psychometric reliability (e.g., Allen et al., 2004; Brandmaier et al., 2018; Tang et al., 2014; Tomarken et al., 1992). Psychometric reliability as used in this manuscript encompasses both the consistency of a feature's estimate within an individual's data obtained within a session (internal consistency as defined by Cronbach (1951); Streiner, 2010; Strube and Newman, 2007) and self-similarity in repeated measurements for individuals across sessions (test-retest reliability as measured by intraclass correlation coefficient proposed first by Fisher (1958); Bartko, 1966; Yen and Lo, 2002). While developmental and clinical studies are particularly fertile contexts for applying individual difference approaches, the changes these populations experience over maturation or clinical course also provide unique challenges in evaluating brain measure psychometric reliability for individual difference analyses (Sanchez-Alonso and Aslin, 2020).

Though issues of measurement reliability in individual differences research are neither new nor unique topics to neuroscience (Elliott et al., 2021; Greene et al., 2022; Kennedy et al., 2022; Kragel et al., 2021; Noble et al., 2021), recent communication about these issues in magnetic resonance imaging (MRI) contexts has led some to declare the entire field of cognitive neuroscience “at a crossroads” in terms of utility for individual differences analyses (“Cognitive neuroscience at the crossroads,” 2022). Is this so? Recent technological and computational advances in electroencephalography (EEG) make it timely to revisit the topic for several reasons. First, EEG has become increasingly cost-effective, mobile, and scalable with engineering advancements that facilitate increased use of this method with larger sample sizes. These technological advancements make individual differences approaches possible and powered to detect a wide range of effects in far more studies moving forward. For example, multiple large-scale, multi-site, or multi-national EEG-based studies (e.g., Healthy Brain and Child Development Study, Wellcome LEAP 1 kD Program, the Autism Biomarkers Consortium of Clinical Trials, Baby Siblings Research Consortium; Jordan et al., 2020; McPartland et al., 2020; Ozonoff et al., 2011) are underway and provide increased opportunities in the near future for performing individual differences analyses with EEG, especially in developmental contexts. There has also been tremendous innovation in the types of EEG features one can extract from the signal that requires consideration of how to optimize their reliability for individual differences research. Moreover, recent shifts in EEG pre-processing and denoising strategies also affect even well-characterized features’ measured reliability profiles across the lifespan. Renewed discussions about EEG measurement reliability and the implications for individual difference study designs and analyses have largely focused on specific populations or EEG measures (e.g., Boudewyn et al., 2018; Clayson, 2020), with far less work looking across types of EEG features or in contexts of change (Becht and Mills, 2020; Foulkes and Blakemore, 2018; Webb et al., 2022).

Here we take a developmental neuroscience perspective to consider progress and new opportunities for parsing the reliability and stability of individual differences in electroencephalography (EEG) measurements across the lifespan. We first conceptually map the different profiles of measurement reliability required for different types of individual difference analyses over the lifespan. Next, we summarize and evaluate the state of the field's empirical knowledge and need for testing measurement reliability, both internal consistency and test-retest reliability, across EEG measures of power, event-related potentials (ERPs), nonlinearity (e.g., entropy, complexity), and functional connectivity across ages. Finally, we highlight two salient challenges and opportunities for change in the research process that may make substantial impact in improving EEG-based individual differences research: 1) standardized, automated pre-preprocessing software for EEG denoising, and 2) empirical metrics of individual data quality. We conclude each topical section with recommendations and resources that individual scientists can implement immediately and in future studies with EEG to improve reproducibility of individual differences findings across the lifespan.

## 2 Mapping profiles of psychometric reliability for individual difference analyses

Individual difference analyses with EEG measures all test questions at the between-person level. They require greater variability in a measure between participants than within a given individual (i.e., one must be more similar to oneself than to other people for a measure). This ensures that researchers can distinguish between individuals with confidence. Thus, testing the psychometric reliability of an EEG feature (the degree to which a measure follows these conditions of self- versus other-similarity) is a critical step in conducting individual difference analyses. Beyond the psychometric reliability constraints however, there is great flexibility in individual difference analyses. They can be performed with a variety of analytic techniques from bivariate correlations to multiple regression frameworks and machine-learning predictive modeling to answer both data-driven and hypothesis-driven research questions (Botdorf et al., 2016; Figueredo et al., 2005; Hounkpatin et al., 2018; Pat et al., 2022; Qin et al., 2014; Sorella et al., 2022). Indeed, here we remain agnostic to the research questions at hand for any individual researcher, but we note the importance of theory, both topical and statistical, in deriving research questions and guiding the research process generally. We hope this manuscript will provide complementary information and guidance about how to best answer the research questions that require individual difference analyses.

It is also important to note briefly that many types of research questions, including some of the earliest and most common in human neuroscience, do not pertain to individual difference analyses at all. Indeed, many commonly-used psychological and neuroscience tasks were designed to *minimize* individual differences in performance and brain activity rather than reveal them (though they can be modified to reveal individual differences if desired; Dai et al., 2019; Soveri et al., 2018). These designs may instead examine differences within-person (e.g., condition differences like eyes-open vs. closed EEG power, most ERP designs with multiple task conditions, even brain-behavior analyses if performed across conditions within-individuals). Reliability considerations for strictly within-person analyses will not be considered here (though see MacDonald and Trafimow (2013) and Trafimow and Rice (2009) for important discourse around those designs). Between-group difference designs may also fall into this category historically, minimizing individual differences to reveal differences between the groups of participants (e.g., EEG-related differences between adults with schizophrenia vs. those without, or between children and adults). Thus, not all of a researcher's questions or designs will need or accommodate considering individual difference-related psychometric reliability profiles.

However, for those studies that do seek to use an individual differences approach, it is important to consider several contextual factors. That is, different contexts require different psychometric profiles of internal consistency and test-retest reliability for valid inferences to be drawn about individual differences. Additional considerations also apply when measuring reliability in contexts of change, including learning, development, and clinical course. Below, we conceptually map this landscape of reliability profiles required for different kinds of individuating EEG markers with illustrative examples. We hope this mapping provides clarity for researchers about what forms of reliability should be assessed with which study designs before conducting particular individual difference analyses of interest.

### 2.1 Patterns of internal consistency reliability

Internal consistency reflects measurement stability within a testing session. As a general rule, individual difference EEG markers for any purpose should be stable features with corresponding high internal consistency scores. That is, there should be high certainty about each individual's estimate for the EEG measure from that testing session. Internal consistency measurements may suffer (i.e., show low stability) for several reasons, including high measurement error, high within-person variability, or low between-person variability (Waltmann et al., 2022). This last condition is non-trivial, as recently noted by several researchers (Hedge et al., 2018; Infantolino et al., 2018), given many standard task designs prioritize within-person differences over between-person differences. Careful task selection (if measuring in a task context) that facilitates differences between participants is therefore critical to facilitate individual difference analyses. Internal consistency should always be evaluated before performing individual difference analyses to ensure both the appropriateness of that analysis and to understand the constraints (bound by internal consistency levels) on potential statistical explanation or prediction in that analysis.

#### 2.1.1 Internally consistent measures of change

We highlight a special case in contexts of change where high within-person variability may nonetheless lead to high internal consistency values if measured appropriately: markers of learning, neural variability, or habituation. For this set of individual difference measures, the stable feature is the change or variability in the EEG signal itself. For example, several lines of clinical research have begun to consider differences in neural variability in conditions like Autism Spectrum Disorder and Schizophrenia (where those with the condition exhibit either increased or decreased variability in EEG measures relative to neurotypical comparisons; MacDonald et al., 2006; Trenado et al., 2018). Individual differences in how quickly brain responses habituate to stimuli provide a related set of inquiries about brain variability in clinical contexts (e.g., Cavanagh et al., 2018; Hudac et al., 2018). Similarly, neural markers of learning (e.g., learning rate) may differ between individuals in meaningful ways (e.g., Waltmann et al., 2022). In each of these cases, although changes are observed and expected over the course of the EEG recording, study design may allow for internal consistency evaluation of the changes. For example, in resting-state EEG, bootstrapped split-half analyses may reveal consistent estimates of the standard deviation of power values between iterations of data-halves. For task paradigms, multiple assessments of the habituation, learning, or variability should be included to enable calculating internal consistency. That is, degree of habituation to tones should be evaluated for two sets of tones, and learning tasks should include at least two rounds of learning to evaluate differences in learning rate or accuracy (e.g., Waltmann et al., 2022). This design may not be possible for all populations or contexts of interest (e.g., long ERP tasks with repeated bouts of learning may not be possible in early developmental contexts, especially for visual paradigms) and may limit when such individual markers of variability and learning are considered accordingly. There may also be interest in measuring the response differences between two conditions as an individual marker of learning or change (i.e., using difference scores as an index of change). Note that two task conditions or ERP components each demonstrating high internal consistency may not necessarily produce a difference score with corresponding high internal consistency (Infantolino et al., 2018; Thigpen et al., 2017). Thus, if difference scores are of interest as potential individual difference markers, the difference score itself must be evaluated for internal consistency rather than the two underlying conditions’ scores. The case of EEG measures of change and variation demonstrate how individual difference analyses do not necessarily preclude within-person variability that may be especially evident in developmental and clinical populations.

### 2.2 Patterns of test-retest reliability

Test-retest reliability reflects stability of markers across testing sessions and time. Unlike internal consistency where higher reliability scores are always more appropriate in individual difference analyses, multiple patterns of test-retest reliability are acceptable depending on the population, analysis purpose, and timescale, as illustrated below.

#### 2.2.1 Low test-retest reliability

Patterns of low test-retest reliability between testing sessions may indeed be valid contexts for a restricted set of individual difference analyses. Namely, if one is interested in associating within-session state-related EEG measures with other in-session state-related measures (e.g., cognition, affective state, etc.), low test-retest reliability may be expected or test-retest reliability may even be impossible to measure. For example, in a decision-making game, participants can change strategy use from session to session, so test-retest reliability of the related EEG measure can be low (each individual will look different from session to session), but there may still be important information within-session about individual differences in degree of strategy use relating to that particular EEG measure. In the context of such brain-cognitive-behavior analyses, the expected stability of the cognitive/affective/behavioral measure is important. If that measure shows high variability across testing administrations within a person, e.g., high influence of state-like (instead of trait-like) contributions, the EEG-measured correlates may similarly show low test-retest reliability while still offering robust relation with the phenomena of interest (e.g., Clarke et al., 2022).

#### 2.2.2 High test-retest reliability

Patterns of high test-retest reliability are desired for several types of individual difference analyses. This profile applies to stable contexts, in which the brain and its relation with physiology, behavior, the environment, or disease state is not expected to vary over time. For example, individual difference analyses in healthy young adults may fit this profile, as may those examining adult EEG features related to trait-like cognitive, affective, or behavioral profiles (e.g., EEG features related to stable temperament or attachment profiles or native language(s) skills). Another case may come from EEG biomarkers of stable disease or disorder characteristics or endophenotypes. Finally, analyses assessing the influence of prior environmental factors or current stable environmental factors on brain-related features may adhere to this reliability profile. For example, how does frequency of emotional abuse in childhood influence young adult EEG-derived neural phenotypes? In each of these cases, there is very low expectation that the brain, and thus, the EEG features, should demonstrate meaningful change from testing session to testing session, and so patterns of high test-retest reliability are expected to infer meaningful individual differences.

#### 2.2.3 High short-term, low long-term test-retest reliability

This final pattern of reliability applies to many contexts of change, including clinical course, developmental change, and intervention targeting. For these populations and/or contexts, change over longer timescales is expected, so they will have low long-term test-retest reliability (though rank order stability measurement may reveal consistent rankings if not estimates over periods of change for some features). Long-term is subjective, of course, and depends on the particular context at hand. For example, low test-retest reliability over many months in infancy may indicate the presence of developmental change rather than lack of reliability for a given EEG measure. Similarly, one may select intervention target EEG measures *because* they exhibit change over time (e.g., plasticity) and thus may be more modifiable than a measure with high stability over the potential intervention ages. Additionally, a reliable marker of clinical severity should not exhibit high long-term test-retest reliability if that encompasses the course of the condition within an individual (instead the marker should change as an individual's status changes with time). Thus, in all of these cases, low long-term test-retest reliability is actually desired. Immediate test-retest stability though can provide confidence that the candidate measure is a reliable indicator of that person's developmental or clinical status in the moment. That is to say, though an EEG feature may change from 9 to 12 months of age, one should still expect high test-retest reliability for that feature if measured same day or the next day. The timescale for determining sufficient measure stability is clinical condition-specific and developmentally-dependent. That is, several weeks between measurements is functionally different in infancy than in adulthood given the respective rates of change in the brain. Still, if a candidate measure's values change within hours of measurement at any age (absent any intervention or clinically-significant change in the interim hours), it will likely have poor utility as an individual difference marker.

### 2.3 Recommendations for individual researchers

We offer the following recommendations that individual researchers can implement in designing studies and planning individual difference analyses from the outset to improve measured reliability profiles.

1) Researchers may use prior literature or pilot testing to ensure individual differences will be elicited by the study paradigm. For examples of researchers evaluating study design changes to optimize individual differences in canonical paradigms, see Dai et al., 2019; Soveri et al., 2018. Ideally, measurement error will be minimized through design and sufficient trials will be planned to ensure stable within-individual estimates can be derived (see Sections 2 and 3 below for guidance in optimizing trial number and trial retention during preprocessing, respectively). Prior test-retest literature may also inform whether the candidate EEG measure(s) of interest fit the reliability profile required for the study context (e.g., if exploring a potential intervention target, does this EEG measure show change within the developmental window of interest?)
2) For designs where learning, habituation, or EEG variability are the features of interest, consider designs that facilitate testing internal consistency of those changes before conducting individual difference analyses (e.g., two blocks of learning, sufficient trials to calculate internal consistency of the variability index like standard deviation, etc.).
3) Finally, researchers should check the assumptions about expected test-retest conditions for their study context and ensure any prior literature or within-lab pilot testing supports those test-retest expectations for the planned individual difference analyses.

## 3 Reliability of EEG measurements across the lifespan

As others have noted before, psychometric reliability is a property of measurement in context rather than the EEG measure itself (e.g., Clayson et al., 2021; Thompson, 2003; Vacha-Haase, 2016). That is, the same EEG measure that may show excellent internal consistency in one population may demonstrate low consistency in a different population or differing consistency when measured in lab-based research settings relative to at-home acquisitions. Reliability may also depend on pre-processing or parameterization of the measure itself (discussed in Section 3 below). Thus, researchers and journals have begun calling for study-specific evaluation and reporting of EEG measure reliability in contexts of individual differences analyses (e.g., Carbine et al., 2021; Clayson, 2020; Clayson et al., 2021a, 2021b, 2019; Clayson and Miller, 2017a, 2017b; Hajcak et al., 2017; Thigpen et al., 2017). We support the momentum to assess and report reliability within each study, and we also believe there is value in looking at the extant reliability literature across ages and types of features for several reasons. First, while measuring internal consistency is technically feasible for each study, measuring test-retest reliability is neither pragmatic nor possible in all cases. Reviewing extant literature may provide guidance (ideally for studies matched for ages, populations, context) and highlight gaps in the field's knowledge that must be filled before measures can be used for some types of individual difference analyses. Second, reviewing reliability findings (both internal consistency and test-retest reliability) across ages and measures provides guidance for new study design and a priori feature selection and parameterization compatible with pre-registration initiatives. We note that drawing on extant literature in these cases does not negate the subsequent need to calculate internal consistency reliability within the study once underway. Third, such a review also provides some information about lifespan change in EEG measure reliability that may not be practical to capture within a single study but may also guide future study design in terms of participant ages or analysis planning. Finally, for several of the more recently introduced EEG features, we hope that reviewing extant literature exploring reliability will provide useful information to guide optimization of these features’ measurement and parameterization (e.g., see nonlinear EEG feature section below).

Therefore, below we evaluate the field's knowledge of reliability for the measures most commonly used with EEG data for in-lab contexts with largely neurotypical populations (unless otherwise indicated). Specifically, we evaluate what is known for power, event-related potentials (ERP), nonlinear, and functional connectivity measures by collating existing studies that have calculated internal consistency and/or test-retest reliability. We summarize the current state of the field's knowledge for each measure with regards to both internal consistency and test-retest reliability for adult populations followed by pediatric populations that experience significant brain change during maturation (here, including infants, toddlers, children, adolescents). Importantly, individual studies have used different reliability metrics and different thresholds (with different degrees of consensus for a given measure) for categorizing reliability results. Consequently, we have elected not to formally make the summary a systematic review. Instead, where there is sufficient literature converging on a single reliability method for an EEG measure, we have focused on reporting comparisons with that particular method for consistency (thus not all studies reporting reliability metrics are included below). This strategy facilitates offering summary statistics about reliability that average across measures and studies. This strategy also lets us focus the review on more contemporary contexts and acquisition setups as more recent studies show increased consensus in reliability measurement and reporting. Moreover, though we report the empirical values of reliability for all studies, to facilitate qualitative conclusions from the collective literature, we have used the following scale of thresholds that we found to be most frequently used in extant literature (reflecting the greatest consensus): poor— values < 0.40; fair—0.40 ≤ values ≤ 0.59; good— 0.60 ≤ values ≤ 0.74; and excellent— values ≥ 0.75 for test- retest reliability (Deuker et al., 2009; Haartsen et al., 2020; Hardmeier et al., 2014; Hatz et al., 2016; Jin et al., 2011; Kuntzelman and Miskovic, 2017). We then provide recommendations for individual actions moving forward for each EEG measure and a final summary across all measures considered.

### 3.1 Power

A common way to quantify EEG oscillatory activity is to compute spectral power, where power is operationalized as the peak signal amplitude squared (Mathalon and Sohal, 2015). There are multiple approaches to characterize EEG power. For example, studies may examine either baseline or task-related power, and quantify absolute power or relative power (the percentage of power in a specific frequency/frequencies relative to total power across all frequencies) (Marshall et al., 2002). Moreover, EEG power is often collapsed across frequencies into canonical frequency bands. These power features can also be combined across hemispheres or frequency bands to form what we refer to as relational power features, like the theta-beta ratio and hemispheric alpha asymmetry features. Finally, contemporary approaches parameterize the power spectrum in terms of periodic (i.e., oscillatory) and aperiodic (e.g., 1/f slope) contributions (e.g., Spectral Parameterization; Donoghue et al., 2020). We consider the internal consistency and test-retest reliability evidence across these types of power measures.

#### 3.1.1 Internal consistency of power measures

**Fig. 1:**
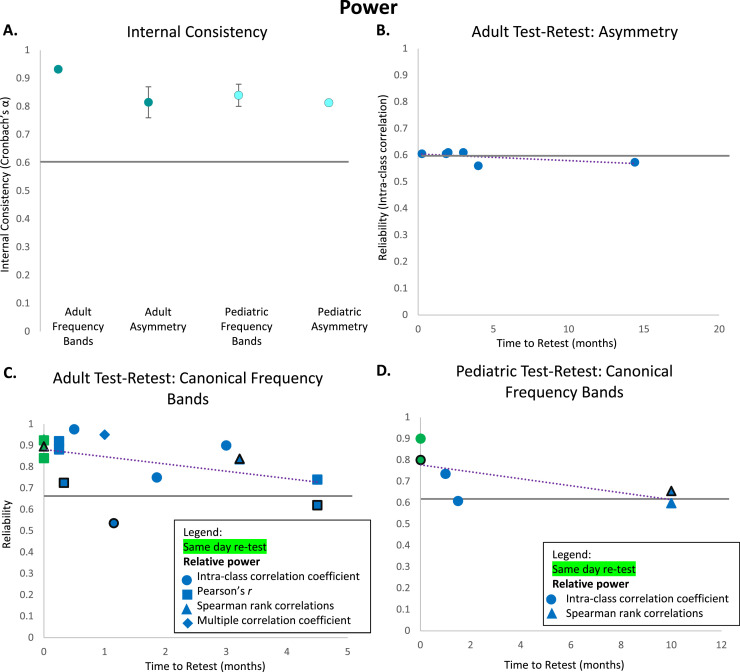
Internal consistency and test-retest reliability of power measures. A: Average internal consistency values calculated using Cronbach's Alpha for adult canonical frequency band power, adult alpha asymmetry, pediatric canonical frequency band power, and pediatric alpha asymmetry. B: Test-retest values calculated using intra-class correlations for each adult alpha asymmetry study based on time between testing sessions (in months). C: Test-retest values calculated using various reliability methods (denoted by different shapes in figure legend) for each adult canonical frequency band power study based on time between testing sessions (in months). D: Test-retest values calculated using various reliability methods (denoted by different shapes in figure legend) for each pediatric canonical frequency band power study based on time between testing sessions (in months). Green markers denote same-day time to retest. Black marker borders denote relative power.

Studies calculating the internal consistency of power measures are surprisingly sparse considering the longevity of EEG power as a measure in the field. Though the studies that do exist suggest power is extremely reliable within testing sessions (Tables 1 and 2). Across power measures, internal consistency values for both adult and pediatric samples are considered excellent (adult: α = 0.92, *n* = 20 measurements, *SE* = 0.01; pediatric: α = 0.83, *n* = 12, *SE* = 0.03). The existing studies evaluate both canonical power frequency bands (adult: α = 0.93, *n* = 18 measurements, *SE* = 0.007; pediatric: α = 0.84, *n* =9 measurements, *SE* = 0.04; Anaya et al., 2021; Burgess and Gruzelier, 1993; Gold et al., 2013; Lund et al., 1995; Rocha et al., 2020, see Fig. 1A) and relational power features like frontal alpha asymmetry (adult: α = 0.82, *n* = 2 measurements, *SE* = 0.06; pediatric: α = 0.81, *n* = 3 measurements, *SE* = 0.007; Allen et al., 2003; Anaya et al., 2021; Gold et al., 2013; Hill et al., 2020; Towers et al., 2008, see Fig. 1A). There is also some evidence that the lowest frequency bands like delta demonstrate lower internal consistency relative to other bands in early development, perhaps because they are quite sensitive to arousal state changes that happen quickly and dramatically during testing in infancy (e.g., Anaya et al., 2021).

**Table 1:** Internal consistency of adult power studies.

Paper	Age(s)	Sample Size	Paradigm	Type of Power	Consistency Measure(s)	Frequency Band	Consistency
Hill et al. (2020)	Not specified	N = 31	Resting-state	Broad frontal asymmetry	Spearman-Brown	Alpha	**0.99**
				High frontal asymmetry	Spearman-Brown	Alpha	**0.99**
Rocha et al. (2020)	Older adults	N = 31	Resting-state	Relative (to nearby frequencies only)	Cronbach's α	Alpha	**0.87**
Towers and Allen (2008)	Undergraduates	N = 204	Resting-state	Frontal asymmetry	Spearman-Brown	Alpha	**0.91**
Burgess and Gruzelier (1993)	18–39 years	N = 24	Resting-state	Absolute	Cronbach's α	Delta	**0.92**
						Theta	**0.95**
						Alpha	**0.95**
						Beta	**0.95**
			Task-related	Absolute	Cronbach's α	Delta	**0.90**
						Theta	**0.94**
						Alpha	**0.90**
						Beta	**0.94**
Allen et al. (2003)	18–45 years	N = 30	Resting-state	Frontal asymmetry	Cronbach's α	Alpha	**0.87**
Gold et al. (2013)	18–50 years	N = 79	Resting-state	Frontal aymmetry	Cronbach's α	Alpha	**0.76**
				Absolute	Cronbach's α	Theta	**0.99**
Lund et al. (1995)	Mean age of 28.5 years	N = 49	Resting-state	Absolute	Cronbach's α	Delta	**0.92**
						Theta	**0.96**
						Alpha	**0.96**
						Beta	**0.95**
				Relative	Cronbach's α	Delta	**0.90**
						Theta	**0.94**
						Alpha	**0.94**
						Beta	**0.90**

**Table 2:** Internal consistency of pediatric power studies.

Paper	Age(s)	Sample Size	Paradigm	Type of Power	Consistency Measure(s)	Frequency Band	Consistency
Hill et al. (2020)	12 months	N = 31	Resting-state	Frontal asymmetry	Spearman-Brown	Alpha	**0.81**
Anaya et al. (2021)	8 months	N = 108	Resting-state	Frontal asymmetry	Cronbach's α	Alpha	**0.80**
				Relative	Cronbach's α	Alpha	**0.89**
						Delta	**0.66**
						Beta	**0.92**
	12 months	N = 71	Resting-state	Frontal asymmetry	Cronbach's α	Alpha	**0.82**
				Relative	Cronbach's α	Alpha	**0.91**
						Delta	**0.73**
						Beta	**0.91**
	18 months	N = 69	Resting-state	Frontal asymmetry	Cronbach's α	Alpha	**0.82**
				Relative	Cronbach's α	Alpha	**0.91**
						Delta	**0.67**
						Beta	**0.96**

#### 3.1.2 Test-retest reliability of canonical power frequency bands

Many studies in adult populations have examined the test-retest reliability of EEG power. Across methods of test-retest evaluation and timescales, baseline EEG power is generally found to be very reliable (Angelidis et al., 2016; Burgess and Gruzelier, 1993; Corsi-Cabrera et al., 2007; Fernández et al., 1993; Keune et al., 2019; McEvoy et al., 2000; Näpflin et al., 2007; Pollock et al., 1991; Rocha et al., 2020; Schmidt et al., 2012; Suárez-Revelo et al., 2015, see Table 3). For example, Ip et al. (2018) have examined test-retest reliability of EEG absolute power using intra- class correlations (*ICC*s) in adults between multiple sessions 20–22 days apart. Adjacent timepoints showed excellent test-retest reliability (*ICC*s = 0.84–.97) in theta, alpha, and beta canonical frequency bands (especially at frontal, midline, and parietal sites). Delta and gamma bands showed more variable test-retest reliability across regions (*ICC*s = 0.30–.87). ICCs across the complete ∼80 day period showed similar results. Studies have also looked at test-retest reliability of EEG power during task paradigms (Fernández et al., 1993; Ip et al., 2018; Keune et al., 2019; McEvoy et al., 2000; Rocha et al., 2020; Salinsky et al., 1991; Schmidt et al., 2012; Suárez-Revelo et al., 2015, see Table 3). For example, McEvoy et al. (2000) found strong correlations between sessions an average of 7 days apart for absolute theta and alpha power during working memory (*r* > 0.9) and psychomotor vigilance (*r* > 0.8) tasks. Several studies have also offered direct comparisons with baseline EEG power reliability. Näpflin et al. (2008) were able to reliably individuate participants over a retest interval of more than one year using absolute alpha peak characteristics during both a working memory task (2008) and from baseline EEG (2007). Though, Ip et al. (2018) found that absolute baseline EEG test-retest reliability was generally higher than absolute task-related EEG power reliability from auditory paradigms within the same individuals over four sessions 20–22 days apart. Taken together, absolute and relative power during tasks appear to be stable across short (several minutes) and long (over one year) intervals in adults, though baseline EEG power may be more reliable than power evoked during at least some task paradigms (Fig. 1C).

**Table 3:** Test-retest reliabilities of adult power studies.

Paper	Age(s) at First Test	Sample Size	Time to Retest	Paradigm	Type of Power	Reliability Measure(s)	Frequency Band	Reliability
McEvoy et al. (2000)	18–29 years	N = 20	1 hour	Resting-state	Absolute	Pearson's *r*	Frontal midline theta	**0.81**
							Posterior theta	**0.85**
							Alpha	**0.91**
				Task-related (WM)	Absolute	Pearson's *r*	Frontal midline theta	**0.96**
							Posterior theta	**0.96**
							Alpha	**0.98**
				Task-related (PVT)	Absolute	Pearson's *r*	Frontal midline theta	**0.94**
							Posterior theta	**0.94**
							Alpha	**0.96**
			7 days	Resting-state	Absolute	Pearson's *r*	Frontal midline theta	**0.79**
							Posterior theta	**0.82**
							Alpha	**0.88**
				Task-related (WM)	Absolute	Pearson's *r*	Frontal midline theta	**0.91**
							Posterior theta	**0.92**
							Alpha	**0.96**
				Task-related (PVT)	Absolute	Pearson's *r*	Frontal midline theta	**0.88**
							Posterior Theta	**0.89**
							Alpha	**0.88**
Angelidis et al. (2016)	18–31 years	N = 41	1 week	Resting-state	Absolute	Pearson's r	Theta	**0.94**
							Beta	**0.90**
							Theta/Beta Ratio	**0.93**
Schmidt et al. (2012)	Mean age of 36.3 years	N = 33	1 week	Resting-state	Absolute	ICC	Frontal alpha	**0.86**
							Central alpha	**0.94**
							Parietal alpha	**0.95**
				Task-related	Absolute	ICC	Frontal alpha	**0.86**
							Central alpha	**0.91**
							Parietal alpha	**0.89**
Suarez-Revelo et al. (2015)	Mean age 23.3 years	N = 15	4–6 weeks	Resting-state	Relative	ICC	Delta	**0.46**
							Theta	**0.76**
							Alpha	**0.75**
							Beta	**0.63**
							Gamma	**0.57**
				Task-related	Relative	ICC	Delta	**0.25**
							Theta	**0.48**
							Alpha	**0.62**
							Beta	**0.52**
							Gamma	**0.32**
Corsi-Cabrera et al. (2007)	18–29 years	N = 6	1 month (total of 9 months)	Resting-state	Absolute	Multiple correlation coefficient *R*	Total power	**0.95**
Pollock et al. (1991)	56–76 years	N = 46	4.5 months	Resting-state	Absolute	Pearson's *r*	Delta	**0.50**
							Theta	**0.81**
							Alpha	**0.84**
							Beta	**0.81**
					Relative	Pearson's *r*	Delta	**0.47**
							Theta	**0.65**
							Alpha	**0.68**
							Beta	**0.68**
Salinsky et al. (1991)	23–52 years	N = 19	5 minutes	Task-related	Absolute	Spearman rank correlations	Delta	**0.90**
							Theta	**0.91**
							Alpha	**0.95**
							Beta	**0.95**
					Relative	Spearman rank correlations	Delta	**0.86**
							Theta	**0.90**
							Alpha	**0.89**
							Beta	**0.93**
			12–16 weeks	Task-related	Absolute	Spearman rank correlations	Delta	**0.81**
							Theta	**0.83**
							Alpha	**0.82**
							Beta	**0.88**
					Relative	Spearman rank correlations	Delta	**0.82**
							Theta	**0.85**
							Alpha	**0.80**
							Beta	**0.88**
Rocha et al. (2020)	Older adults	N = 31	10 days	Resting-state	Relative (to nearby frequencies only)	Pearson's *r*	Alpha	**0.62**
				Task-related	Relative (to nearby frequencies only)	Pearson's *r*	Alpha	**0.83**
Burgess and Gruzelier (1993)	18–39 years	N = 24	40 minutes	Resting-state	Absolute	Pearson's *r*	Delta	**0.70**
							Theta	**0.86**
							Alpha	**0.91**
							Beta	**0.89**
Allen et al. (2003)	18–45 years	N = 26	8 weeks	Resting-state	Frontal asymmetry	ICC	Alpha	**0.61**
		N = 15	16 weeks	Resting-state	Frontal asymmetry	ICC	Alpha	**0.56**
Pathania et al. (2021)	Undergraduates	N = 60	30 minutes	Resting-state	Spectral slope (LMER) feature	ICC	Power spectral density	**0.91**
					Aperiodic slope (SpecParam)	ICC	Power spectral density	**0.86**
Keune et al. (2019)	18–75 years	N = 10	2 weeks	Resting-state/Task-related	Absolute	ICC	Theta/beta ratio	**0.96**
							Theta	**0.98**
							Beta	**0.97**
Gold et al. (2013)	18–50 years	N = 79	3 months	Resting-state	Frontal asymmetry	ICC	Alpha	**0.61**
					Absolute	ICC	Theta	**0.90**
Vuga et al. (2006)	19–39 years	N = 99	1.2 years	Resting-state	Lateral frontal asymmetry	ICC	Alpha	**0.60**
					Mid-frontal asymmetry	ICC	Alpha	**0.54**
					Parietal asymmetry	ICC	Alpha	**0.58**
Koller-Schlaud et al. (2020)	Mean age of 27 years	N = 23	7 days	Task-related	Frontomedial asymmetry	ICC	Alpha	**0.52**
					Frontolateral asymmetry	ICC	Alpha	**0.70**
					Parietomedial aymmetry	ICC	Alpha	**0.71**
					Parietolateral asymmetry	ICC	Alpha	**0.49**
Metzen et al. (2021)	20–70 years	N = 541	56.7 days	Resting-state	Absolute	ICC	Alpha	**0.75**
					Frontal asymmetry	ICC	Alpha	**0.56**
					Parietal asymmetry	ICC	Alpha	**0.65**
Tenke et al. (2018)	18+ years	N = 46	12 years	Resting-state	Absolute (CSD-fPCA)	Spearman Brown	Alpha	**0.92**
					Posterior asymmetry	Spearman Brown	Alpha	**0.73**

In contrast, relational power measures, formed by relating power in specific frequency bands or over specific scalp topography, have shown more variable reliability, ranging from fair to excellent in adults. For example, the theta beta ratio measured in resting-state EEG has shown consistently excellent test-retest reliability on the scale of weeks in younger and older adults (*r* = 0.93 in young adults (Angelidis et al., 2016); *ICC* = 0.96 in older adults (Keune et al., 2019)). Additionally, alpha asymmetry has shown fair to good reliability in adults when time to retest spans from one week to over a decade later (*ICC = 0*.61 at 1 week (Koller-Schlaud et al., 2020); *ICC = 0*.61 at 8 weeks and *ICC = 0*.56 at 16 weeks (Allen et al., 2003); *ICC = 0*.61 at 56 days (Metzen et al., 2021); *ICC = 0*.61 at 3 months (Gold et al., 2013); *rSB =0*.73 at 12 years (Tenke et al., 2018), see Fig. 1B). Overall, studies examining frequency band power in adults indicate adequate reliability across most bands and relational band measures over time periods longer than a year.

Few studies to date have examined test-retest reliability of EEG power bands in pediatric populations, and those that do have been conducted only for baseline EEG with older children and adolescents (Table 4). Gasser et al. (1985) used spearman rank correlations to show that absolute and relative baseline power in most frequency bands and locations showed similar patterns of test-retest reliability in typically-developing peri-adolescents over 10 months (mean values for ρ = 0.58–.80 for absolute power; ρ = 0.47–.80 for relative power). Similarly, Winegust et al. (2014) have shown that baseline absolute frontal alpha power demonstrates borderline excellent test-retest reliability (*ICC*s = 0.73–.74) over a one-month interval in typically developing adolescents. Several pediatric studies have also examined whether power test-retest reliability differs between typically-developing and clinical populations. Fein et al. (1983) found that same-day test-retest reliability for power was good-excellent for both dyslexic and typically- developing children (*ICC*s > 0.70). Similarly, Levin et al. (2020) found that both typically- developing and autistic children demonstrated excellent reliability of baseline total power over up to several weeks when processed with standardized software (here, HAPPE and BEAPP software; typically-developing group ICC = 0.86; autistic group ICC = 0.81). However, a recent paper by Webb et al. (2022) found that typically-developing children demonstrated only fair reliability across power bands over a period of six weeks, while autistic children demonstrated good reliability over the same time period (typically-developing group ICC = 0.54; autistic group ICC = 0.68). Several of these studies have also noted higher ICCs for absolute compared to relative baseline power in development (Fein et al., 1983; Gasser et al., 1985). Relatedly, there is limited knowledge about the test-retest reliability of relational power features (e.g., alpha asymmetry, theta-beta ratio). For example, Vincent et al. (2021) and Anaya et al. (2021) have both found that frontal alpha asymmetry scores were only weakly stable across infancy and early childhood, whether measured as correlated values or rank orders. Delta-beta ratios demonstrated similar age-related changes during this early developmental window (Anaya et al., 2021). In sum, these studies suggest high reliability of canonical frequency band power (especially absolute power) in both healthy and clinical pediatric populations in childhood through adolescence (Fig. 1D).

**Table 4:** Test-retest reliabilities of pediatric power studies.

Paper	Age(s) at First Test	Sample Size	Time to Retest	Paradigm	Type of Power	Reliability Measure(s)	Frequency Band	Reliability
Winegust et al. (2014)	Mean age of 15.9 years	N = 9	1 month	Resting-state	Absolute	ICC	Left mid-frontal alpha	**0.74**
							Right mid-frontal alpha	**0.73**
					Absolute	Pearson's *r*	Left mid-frontal alpha	**0.74**
							Right mid-frontal alpha	**0.73**
Levin et al. (2020)	Mean age of 6.6 years (TD group)	N = 26	Median 6 days	Resting-state	Relative	ICC	Total power	**0.86**
					Spectral offset (SpecParam)	ICC	Power spectral density (PSD)	**0.48**
					Aperiodic slope (SpecParam)	ICC	Power spectral density (PSD)	**0.28**
					Number of peaks (SpecParam)	ICC	Power spectral density (PSD)	**0.02**
					Largest alpha peak: center (SpecParam)	ICC	Alpha	**0.70**
					Largest alpha peak: amplitude (SpecParam)	ICC	Alpha	**0.86**
					Largest alpha peak: bandwidth (SpecParam)	ICC	Alpha	**0.42**
	Mean age of 8 years (ASD group)	N = 21	Median 6 days	Resting-state	Relative	ICC	Total power	**0.81**
					Spectral offset (SpecParam)	ICC	Power spectral density (PSD)	**0.53**
					Aperiodic slope (SpecParam)	ICC	Power spectral density (PSD)	**0.70**
					Number of peaks (SpecParam)	ICC	Power spectral density (PSD)	**0.23**
					Largest alpha peak: center (SpecParam)	ICC	Alpha	**0.62**
					Largest alpha peak: amplitude (SpecParam)	ICC	Alpha	**0.83**
					Largest alpha peak: bandwidth (SpecParam)	ICC	Alpha	**0.34**
Gasser et al. (1985)	10–13 years	N = 26	10 months	Resting-state	Absolute	Spearman rank correlations	Delta	**0.59**
							Theta	**0.70**
							Alpha	**0.76**
							Beta	**0.62**
					Relative	Spearman rank correlations	Delta	**0.47**
							Theta	**0.63**
							Alpha	**0.76**
							Beta	**0.76**
Fein et al. (1983)	10–12 years	N = 32	4–5 hours	Resting-state	Absolute	ICC	Total power	**> 0.90**
					Relative	ICC	Total power	**0.70 - 0.90**
Vincent et al. (2021)	5 months, 7 months, or 12 months	N = 149	24–31 months	Resting-state	FAAln	Pearson's *r*	Alpha	**-0.02**
					FAAratio	Pearson's *r*	Alpha	**-0.02**
					FAAlnratio	Pearson's *r*	Alpha	**-0.07**
					FAAlnrel	Pearson's *r*	Alpha	**0.36**
Anaya et al. (2021)	8 months	N = 43–89	4 months	Resting-state	Frontal asymmetry	Pearson's *r* (rank-order stability)	Alpha	**0.09**
					Delta-Beta coupling	Pearson's *r* (rank-order stability)	Delta/Beta Ratio	**-0.06**
			6 months	Resting-state	Frontal asymmetry	Pearson's *r* (rank-order stability)	Alpha	**-0.19**
					Delta-Beta coupling	Pearson's *r* (rank-order stability)	Delta/Beta Ratio	**0.08**
			10 months	Resting-state	Frontal asymmetry	Pearson's *r* (rank-order stability)	Alpha	**0.27**
					Delta-Beta coupling	Pearson's *r* (rank-order stability)	Delta/Beta Ratio	**-0.05**
Webb et al. (2022)	6–11.5 years (TD Group)	N = 119	6 weeks	Resting-state	Absolute	ICC	Delta	**0.39**
							Theta	**0.51**
							Beta	**0.68**
							Alpha	**0.67**
							Gamma	**0.45**
					Aperiodic slope (SpecParam)	ICC	2–50 Hz	**0.54**
	6–11.5 years (ASD Group)	N = 280	6 weeks	Resting-state	Absolute	ICC	Delta	**0.66**
							Theta	**0.68**
							Beta	**0.75**
							Alpha	**0.73**
							Gamma	**0.56**
					Aperiodic slope (SpecParam)	ICC	2–50 Hz	**0.59**

#### 3.1.3 Test-retest reliability of periodic and aperiodic power spectrum features

Studies have also used the Spectral Parameterization (i.e., SpecParam, formerly Fitting Oscillations and One-Over-F (FOOOF)) and other algorithms developed recently (Donoghue et al., 2020; Wen and Liu, 2016) to characterize the EEG power spectrum in terms of periodic and aperiodic features (e.g., Ostlund et al., 2021). This direction is especially important given the emerging recognition and exploration of differences in functional significance and neural underpinnings for the periodic versus aperiodic power spectrum components (Colombo et al., 2019; Demanuele et al., 2007; He et al., 2010; McDonnell and Ward, 2011; Podvalny et al., 2015). Fortunately, though this approach is quite new, several studies have already investigated the reliability of periodic/aperiodic power spectrum features with promising preliminary results. In adults, the aperiodic slope shows excellent test-retest reliability on the same day with two different methods of calculation, linear mixed-effects regression and SpecParam (LMER *ICC*s = 0.85–.95, SpecParam *ICC*s = 0.78–.93; Pathania et al., 2021). Using a different approach, Demuru and Fraschini (2020) investigated how SpecParam features can identify participants from a large dataset of *n* = 109 of baseline EEG recordings. The SpecParam spectral offset and aperiodic slope features both performed very well as discriminators. In pediatric populations, Levin et al. (2020) found variable reliability estimates across baseline EEG SpecParam measures between sessions of about one week apart in typically-developing and autistic children. For example, aperiodic slope demonstrated variable reliability estimates between participant groups, with good reliability in the autism group (*ICC* = 0.70) and poor reliability in the typically-developing group (*ICC* = 0.28). Over a longer period of six weeks, Webb et al. (2022) found that aperiodic slope had fair reliability across both a typically-developing group (*ICC = 0*.54) and autism group (*ICC = 0*.59). Further, Levin et al. (2020) found that other features had poor reliability across all children, including the number of spectrum peaks. Thus, some SpecParam features may be more appropriate for biomarker/individual difference investigations than others over development. Overall, the studies reviewed here in pediatric and adult samples suggest that EEG aperiodic features, such as aperiodic slope and offset, may be a reliable source of interindividual variation.

#### 3.1.4 Power measurement recommendations

Given the reliability studies conducted from childhood through adulthood so far, we offer the following recommendations. 1) Power measured in canonical frequency bands typically has sufficient internal consistency and test-retest reliability from childhood through adulthood to be considered for any individual difference study design. There is some evidence that measuring power during baseline/resting-state conditions produces more reliable estimates than measuring power during task paradigms, though in studies to date, power in both contexts is adequately reliable for individual difference analyses. 2) Relational power features like frontal alpha asymmetry have shown excellent internal consistency in adult and pediatric samples but display only fair-good test-retest reliability in adulthood and inadequate test-retest reliability in studies conducted in infancy through early childhood. 3) Contemporary approaches characterizing the power spectrum through periodic and aperiodic features (e.g., Spectral Parameterization, IRASA) show promise for reliable measurement but further testing should be undertaken in both pediatric and adult populations to explore optimizing these measurements’ reliability for individual difference assessments. Tutorials are available to guide users through applying spectral parameterization methods to their data (e.g., Ostlund et al., 2022; Voytek Lab (https://fooof-tools.github.io/fooof/auto_tutorials/index.html); Wilson and Cassani (https://neuroimage.usc.edu/brainstorm/Tutorials/Fooof)). 4) Very little is reported on internal consistency and test-retest reliability of any power measurements in infants and young children. Though test-retest reliability is especially challenging to measure with fidelity at the youngest ages when change is most rapid, researchers must begin measuring and reporting internal consistency values for their power measurements at these ages with available tools before using them for individual difference analyses.

### 3.2 Event-related potentials

Event-related potentials (ERPs) have been used extensively across the lifespan as a temporally-sensitive measure of task-evoked brain activity. Analyses often break ERPs down into components, distinct deflections in the ERP waveform characterized by location on the scalp, polarity, timing post-stimulus (i.e., latency), and sometimes task context. These different ERP components relate to specific cognitive, affective, and perceptual processes in the brain, so ERP components are being used in a variety of individual difference study designs, especially in clinical populations (e.g., Beker et al., 2021; Cremone-Caira et al., 2020; Webb et al., 2022; for best practices in using ERPs in clinical populations, see (Kappenman and Luck, 2016). Fortunately, there is a large body of work evaluating the internal consistency and test-retest reliability profiles of different ERP measurements (for examples, see recent meta-analysis from Clayson (2020) on reliability of the error-related negativity (ERN) and this thorough examination of factors influencing reliability of ERPs from Boudewyn et al. (2018)). We summarize this literature with respect to peak amplitude, mean amplitude, and latency to peak amplitude measurements, the most commonly assessed ERP measures (though see Clayson et al. (2013) and Luck and Gaspelin (2017) for arguments against using peak amplitude for reliability reasons).

#### 3.2.1 Internal consistency of ERPs

**Fig. 2:**
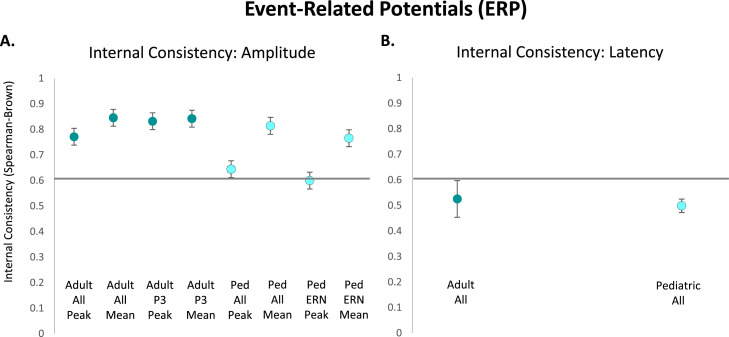
Internal consistency of event-related potentials (ERP). A: Average internal consistency values calculated using the Spearman-Brown Formula for all adult ERP peak amplitudes, all adult ERP mean amplitudes, adult P3 peak amplitudes, adult P3 mean amplitudes, all pediatric ERP peak amplitudes, all pediatric ERP mean amplitudes, pediatric ERN peak amplitudes, and pediatric ERN mean amplitudes. B: Average internal consistency values calculated using the Spearman-Brown Formula for all adult ERP latencies (to peak amplitudes) and all pediatric ERP latencies.

Fewer internal consistency studies were identified relative to test-retest reliability studies across the lifespan that used consistent reliability metrics (see Table 5, Table 6, Table 7, Table 8). Available evidence (Cassidy et al., 2012; Hämmerer et al., 2012; D.M. Olvet and Hajcak, 2009a; Sandre et al., 2020; Walhovd and Fjell, 2002) suggests ERP peak amplitude measurements in adults across studies usually demonstrate excellent internal consistency ^(^*^r^SB ^= 0^*^.77,^ *^n^* ^= 31 measurements,^ *^SE^* ^= 0.03;^ Fig. 2A^).^ Meanwhile, adult mean amplitude studies (Bresin and Verona, 2021; Cassidy et al., 2012; Foti et al., 2013; Hajcak et al., 2017; Levinson et al., 2017; Meyer et al., 2013; Pontifex et al., 2010; Sandre et al., 2020; Xu and Inzlicht, 2015) indicated even higher levels of excellent internal consistency across all ERPs (*^r^SB ^= 0^*^.85,^ *^n^* ^= 22 measurements,^ *^SE^* ^= 0.02;^ Fig. 2A). ^The P3, a component^ related to attention and working memory, was most commonly evaluated across adult peak amplitude studies (Cassidy et al., 2012; Hämmerer et al., 2012; Walhovd and Fjell, 2002) and adult mean amplitude studies (Bresin and Verona, 2021; Cassidy et al., 2012), demonstrating excellent internal consistency for both types of amplitude measurement (peak: *rSB* = 0.83, *n* = 9 measurements, *SE* = 0.04; mean: *rSB* = 0.84, *n* = 8 measurements, *SE* = 0.04; Fig. 2A).

**Table 5:** Internal consistency of adult ERP peak amplitude and mean amplitude studies.

Paper	Age(s)	Sample Size	Consistency Measure	ERP Measure(s)	Consistency
Cassidy et al. (2012)	19–35 years	N = 25	Spearman-Brown	P1 Peak	**0.73**
				N1 (P08) Peak	**0.88**
				N1 (P07) Peak	**0.89**
				P3a Peak	**0.93**
				P3a Difference Peak	**0.66**
				P3b Peak	**0.73**
				P3b Difference Peak	**0.63**
				ERN Peak	**0.64**
				ERN Difference Peak	**0.72**
				Pe Peak	**0.88**
				Pe Difference Peak	**0.89**
				P400 Peak	**0.87**
				N170 Peak	**0.81**
				ERN Peak-to-Peak	**0.51**
				ERN Difference Peak-to-Peak	**0.44**
				P3a Mean	**0.9**
				P3a Difference Mean	**0.70**
				P3b Mean	**0.68**
				P3b Difference Mean	**0.71**
				Pe Mean	**0.76**
				Pe Difference Mean	**0.85**
				P400 Mean	**0.88**
				Area Under the P3a	**0.89**
				Area Difference P3a	**0.73**
				Area Under the P3b	**0.63**
				Area Difference P3b	**0.74**
				Area Under the Pe	**-0.08**
				Area Difference Pe	**0.84**
				Area Under the P400	**0.73**
Levinson et al. (2017)	Undergraduates	N = 59	Spearman-Brown	FN Mean	**0.80**
				RewP Mean	**0.86**
			Cronbach's α	FN Mean	**0.82**
				RewP Mean	**0.86**
Walhovd & Fjell (2002)	Mean age of 56.1 years	N = 59	Spearman-Brown	P3 (Pz) Peak	**0.92**
				P3 (Cz) Peak	**0.93**
				P3 (Fz) Peak	**0.84**
Hämmerer et al. (2012)	Younger adults (mean age = 24.27 years)	N = 47	Spearman-Brown	Go-P3 Peak	**0.96**
	Older adults (mean age = 71.24 years)	N = 47	Spearman-Brown	Go-P3 Peak	**0.89**
Hajcak et al. (2017)	Not specified	N = 53	Spearman-Brown	ERN Mean	**0.75**
			Cronbach's α	ERN Mean	**0.75**
Meyer et al. (2013)	Mean age of 19.14 years	N = 43	Cronbach's α	ERN Mean	**0.70**
D.M. Olvet and Hajack (2009)	Undergraduates	N = 45	Spearman-Brown	CRN Peak	**0.98**
				ERN Peak	**0.86**
				ERN-CRN Difference Peak	**0.80**
				Area Under the CRN	**0.98**
				Area Under the ERN	**0.86**
				Area Difference ERN-CRN	**0.71**
				Area Under the Pe	**0.87**
Bresin and Verona (2021)	Mean age of 29 years	N = 55	Spearman-Brown	P3 Incongruent Mean	**0.92**
				P3 Congruent Mean	**0.93**
				P3 No-Go Mean	**0.95**
				P3 Go Mean	**0.95**
				ERN Mean	**0.80**
				CRN Mean	**0.92**
				Pe Mean	**0.78**
				Pc Mean	**0.94**
Foti et al. (2013)	18–65 years (Healthy Individuals)	N = 52	Cronbach's α	ERN Mean Flanker	**0.86**
				ΔERN Mean Flanker	**0.84**
				Pe Mean Flanker	**0.81**
				ΔPe Mean Flanker	**0.83**
				ERN Mean Picture/Word Task	**0.41**
				ΔERN Mean Picture/Word Task	**0.69**
				Pe Mean Picture/Word Task	**0.66**
				ΔPe Mean Picture/Word Task	**0.79**
	28–68 years (Patients with Psychotic Illness)	N = 84	Cronbach's α	ERN Mean Flanker	**0.63**
				ΔERN Mean Flanker	**0.48**
				Pe Mean Flanker	**0.75**
				ΔPe Mean Flanker	**0.73**
				ERN Mean Picture/Word Task	**0.35**
				ΔERN Mean Picture/Word Task	**0.40**
				Pe Mean Picture/Word Task	**0.28**
				ΔPe Mean Picture/Word Task	**0.39**
Pontifex et al. (2010)	18–25 years and 60–73 years	N = 83	Cronbach's α	ERN Mean	**0.96**
				Pe Mean	**at least 0.90**
Sandre et al. (2020)	Mean age of 20.1 years	N = 263	Spearman-Brown	ERN Peak (Cz)	**0.80**
				ERN Peak (FCavg)	**0.76**
				CRN Peak (Cz)	**0.82**
				CRN Peak (FCavg)	**0.72**
				ERN Peak-to-Peak (Cz)	**0.75**
				ERN Peak-to-Peak (FCavg)	**0.67**
				CRN Peak-to-Peak (Cz)	**0.54**
				CRN Peak-to-Peak (FCavg)	**0.47**
				ERN Mean (Cz)	**0.81**
				ERN Mean (FCavg)	**0.78**
				CRN Mean (Cz)	**0.97**
				CRN Mean (FCavg)	**0.97**
			Cronbach's α	ERN Peak (Cz)	**0.73**
				ERN Peak (FCavg)	**0.63**
				CRN Peak (Cz)	**0.77**
				CRN Peak (FCavg)	**0.68**
				ERN Peak-to-Peak (Cz)	**0.74**
				ERN Peak-to-Peak (FCavg)	**0.64**
				CRN Peak-to-Peak (Cz)	**0.80**
				CRN Peak-to-Peak (FCavg)	**0.66**
				ERN Mean (Cz)	**0.63**
				ERN Mean (FCavg)	**0.57**
				CRN Mean (Cz)	**0.75**
				CRN Mean (FCavg)	**0.70**
Xu and Inzlicht (2015)	Mean age of 19.2 years	N = 39	Cronbach's α	ERN Mean	**0.59**
				CRN Mean	**0.97**
				ΔERN Mean	**0.74**
				Pe Mean	**0.94**
				ΔPe Mean	**0.92**

**Table 6:** Internal consistency of pediatric ERP peak amplitude and mean amplitude studies.

Paper	Age(s)	Sample Size	Consistency Measure	ERP Measure(s)	Consistency
Jetha et al. (2021)	Kindergarten - 1st grade	N = 110	Spearman Rho (rank order stability)	P1 Peak (O1)	**0.59**
				P1 Peak (O2)	**0.64**
				P1 Peak (Oz)	**0.69**
				N170 Peak (P7)	**0.67**
				N170 Peak (P8)	**0.54**
				VPP Peak (Fz)	**0.59**
				VPP Peak (FC1)	**0.62**
				VPP Peak (FC2)	**0.56**
				VPP Peak (Cz)	**0.65**
Meyer et al. (2014)	8–13 years	N = 44	Spearman-Brown	ERN Peak (Fz) Flanker	**0.67**
				ERN Peak (Cz) Flanker	**0.85**
				ERN Peak (Fz) Go-NoGo	**0.38**
				ERN Peak (Cz) Go-NoGo	**0.50**
Hämmerer et al. (2012)	Children (mean age = 10.15 years)	N = 45	Spearman-Brown	Go-P3 Peak	**0.81**
	Adolescents (mean age = 14.38 years)	N = 46	Spearman-Brown	Go-P3 Peak	**0.91**
Luking et al. (2017)	8–14 years	N = 177	Spearman-Brown	ERN Gain Mean	**0.85**
				ERN Loss Mean	**0.86**
				ERN Difference (Gain-Loss) Mean	**0.36**
Morales et al. (2022)	4–9 years	N = 326	Spearman-Brown	ERN Correct Mean	**0.96**
				ERN Error Mean	**0.80**
				Pe Correct Mean	**0.98**
				Pe Error Mean	**0.89**
Pontifex et al. (2010)	8–11 years	N = 83	Cronbach's α	ERN Mean	**at least 0.90**
				Pe Mean	**at least 0.90**

**Table 7:** Internal consistency of adult ERP latencies (to peak amplitudes) studies.

Paper	Age(s)	Sample Size	Consistency Measure	ERP Measure(s)	Consistency
Cassidy et al. (2012)	19–35 years	N = 25	Spearman-Brown	P1	**0.70**
				N1 (O2)	**0.86**
				N1 (P07)	**0.93**
				P3a	**0.38**
				P3a Difference	**0.34**
				P3b	**0.01**
				P3b Difference	**0.03**
				ERN	**0.39**
				ERN Difference	**0.15**
				Pe	**0.52**
				Pe Difference	**0.30**
				P400	**0.05**
				N170	**0.87**
Walhovd & Fjell (2002)	Mean age 56.1 years	N = 59	Spearman-Brown	P3 (Pz)	**0.77**
				P3 (Cz)	**0.79**
				P3 (Fz)	**0.77**
D.M. Olvet and Hajack (2009)	Undergraduates	N = 45	Spearman-Brown	CRN	**0.86**
				ERN	**0.56**
				ERN-CRN Difference	**0.71**

**Table 8:** Internal consistency of pediatric ERP latencies (to peak amplitudes) studies.

Paper	Age(s)	Sample Size	Consistency Measure	ERP Measure(s)	Consistency
Jetha et al. (2021)	Kindergarten - 1st grade	N = 110	Spearman Rho (rank order stability)	P1 (O1)	**0.57**
				P1 (O2)	**0.36**
				P1 (Oz)	**0.37**
				N170 (P7)	**0.52**
				N170 (P8)	**0.53**
				VPP (Fz)	**0.55**
				VPP (FC1)	**0.55**
				VPP (FC2)	**0.53**
				VPP (Cz)	**0.51**

With regard to pediatric studies in childhood and adolescence (Hämmerer et al., 2012; Jetha et al., 2021; Meyer et al., 2014), the internal consistency of peak amplitudes across all ERPs was considered good (*rSB* = 0.65, *n* = 15 measurements, *SE* = 0.04; Fig. 2A). Meanwhile, studies measuring mean amplitude across all ERPs in childhood and adolescence (Luking et al., 2017; Morales et al., 2022; Pontifex et al., 2010) indicated excellent internal consistency (*rSB* = 0.81, *n* = 7 measurements, *SE* = 0.08; Fig. 2A), outperforming peak amplitude measurement in pediatric samples. However, it is important to note the limited number of studies evaluating mean amplitude internal consistency compared to peak amplitude internal consistency in pediatric samples. The peak amplitude of the ERN component, related to error processing, was most commonly measured in pediatric samples for both peak amplitude (Meyer et al., 2014) and mean amplitude (Luking et al., 2017; Morales et al., 2022; Pontifex et al., 2010), demonstrating good internal consistency across studies of ERN peak amplitude (*rSB* = 0.60, *n* = 4 measurements, *SE* = 0.10; Fig. 2A) and excellent internal consistency across studies of ERN mean amplitude (*rSB* = 0.77, *n* = 5 measurements, *SE* = 0.10; Fig. 2A). Note, once again, the literature is still quite limited in evaluating the ERN using either peak or mean amplitude, necessitating additional evidence to more confidently draw conclusions regarding its reliability. Collectively, evidence to date suggests that mean ERP amplitude measurements across all ages and peak ERP amplitude in adulthood demonstrate excellent internal consistency, while peak ERP amplitude in pediatric samples indicates lower internal consistency. Measurements of mean ERP amplitude thus seem the best candidates to target for individual difference analyses across the lifespan.

Internal consistency of ERP latency measurements is not as well-characterized. Latencies are typically calculated as latency-to-peak amplitude. Based on the only three adult papers identified (Cassidy et al., 2012; D.M. Olvet and Hajcak, 2009a; Walhovd and Fjell, 2002), the internal consistency of ERP latencies was fair (*rSB* = 0.53, *n* = 19 measurements, *SE* = 0.07; Fig. 2B) across components. In pediatric samples, we identified only one paper that found fair ERP latency internal consistency (*rSB* = 0.50, *n* = 9 measurements, *SE* =0.03; Jetha et al., 2021; Fig. 2B). Acquisition set-ups may affect latency measurements in more ways than amplitude measurements (which are usually extracted within a temporal window whereas latency measurements are not). For example, the precision of presentation timing may particularly impact latency measurements, though recent hardware solutions (e.g., the Cedrus Stimtracker) may improve latency reliability measurements relative to historical measurements. Further testing is required across the lifespan to evaluate the consistency of ERP latency measurements.

#### 3.2.2 Test-retest reliability of ERPs

**Fig. 3:**
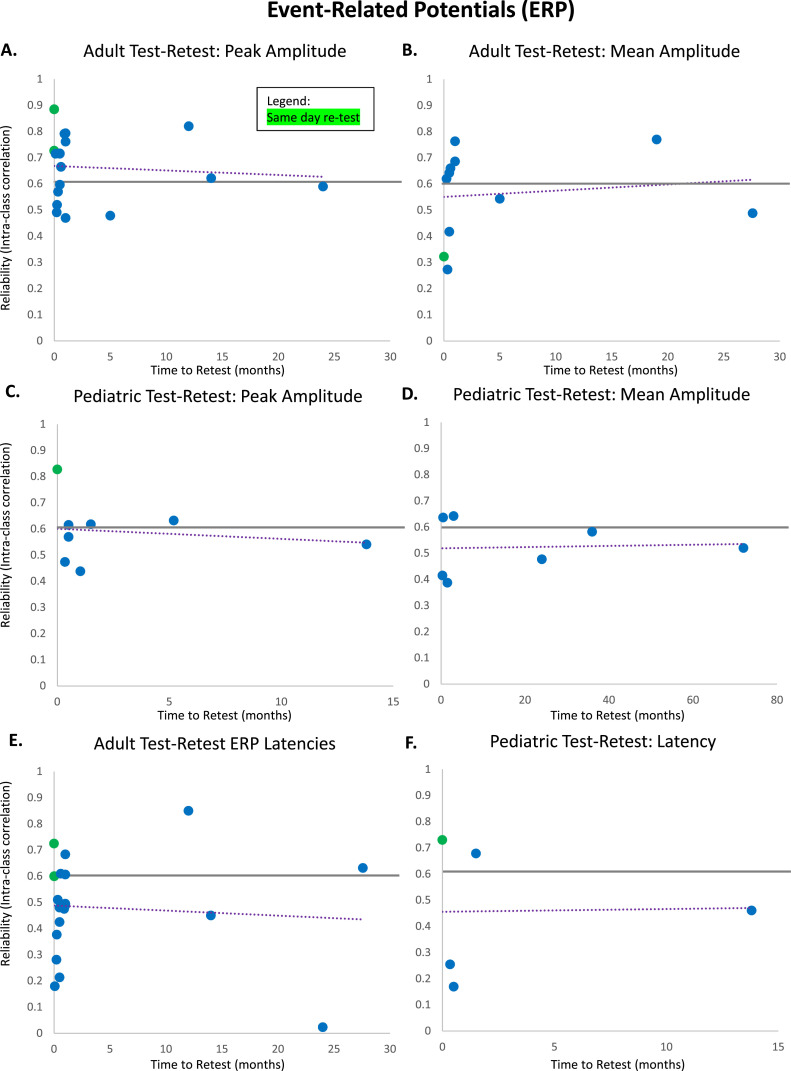
Internal consistency and test-retest reliability of event-related potentials (ERP). A: Average internal consistency values calculated using the Spearman-Brown Formula for all adult ERP peak amplitudes, adult P3 peak amplitudes, all pediatric ERP peak amplitudes, and pediatric ERN peak amplitudes. B: Average internal consistency values calculated using the Spearman-Brown Formula for all adult ERP latencies (to peak amplitudes) and all pediatric ERP latencies. C: Test-retest values calculated using intra-class correlations for each adult ERP amplitude study based on time between testing sessions (in months). D: Test-retest values calculated using intra-class correlations for each adult ERP latency study based on time between testing sessions (in months). E: Test-retest values calculated using intra-class correlations for each pediatric ERP amplitude study based on time between testing sessions (in months). F: Test-retest values calculated using intra-class correlations for each pediatric ERP latency study based on time between testing sessions (in months). Green markers denote same-day time to retest.

There is a robust test-retest reliability literature for ERPs across the lifespan, so to compare and summarize across results, we focus here on studies that calculated intraclass correlation coefficients (ICC) as the measure of test-retest reliability for both peak amplitude and mean amplitude (see Table 9, Table 10, Table 11, Table 12). Intraclass correlation coefficients are the most commonly used measure of reliability across ERP studies, providing the largest number of studies for direct comparison. Further, ICCs were the predominant measure used across more recent ERP reliability studies, providing the most current reliability estimates in the literature*.* Across all adult papers that calculated ICCs for ERP peak amplitude (Brunner et al., 2013; Cassidy et al., 2012; Fallgatter et al., 2001; Hall et al., 2006; Hämmerer et al., 2012; Kinoshita et al., 1996; Lew et al., 2007; Lin et al., 2020; Olvet and Hajcak, 2009a; Rentzsch et al., 2008; Sandre et al., 2020; Segalowitz et al., 2010; Sinha et al., 1992; Suchan et al., 2019; Taylor et al., 2016; Thesen and Murphy, 2002; Wang et al., 2021; Weinberg and Hajcak, 2011), reliability was generally good (*ICC* = 0.65, *n* = 109 measurements, *SE* = 0.02), though over a third of the studies did not report ICCs greater than the commonly-used threshold of 0.6 for inclusion in individual difference studies (7/20 studies). The effect of time to retest across studies with adults was also considered (range = 20 min – 2 years; see Fig. 3A). Meanwhile, across all adult studies that calculated ICCs for mean amplitude (Cassidy et al., 2012; Chen et al., 2018; Hall et al., 2006; Huffmeijer et al., 2014; Larson et al., 2010; Levinson et al., 2017; Light and Braff, 2005; Malcolm et al., 2019; Sandre et al., 2020; Taylor et al., 2016), reliability was only fair (*ICC* = 0.56, *n* = 44 measurements, *SE* = 0.03), regardless of time to retest (range = 7 hours – 2.3 years; see Fig. 3B). Further, the overall test-retest reliability across ERP peak amplitude measurements in studies with pediatric populations (Beker et al., 2021; Hämmerer et al., 2012; Jetha et al., 2021; Kompatsiari et al., 2016; Lin et al., 2020; Segalowitz et al., 2010; Taylor et al., 2016; Webb et al., 2022) achieved good reliability (*ICC* = 0.60, *n* = 73 measurements, *SE* = 0.02), without much change as a function of time to retest (range = 30 min – 1.15 years; see Fig. 3C). Meanwhile, pediatric ERP mean amplitude studies (Cremone-Caira et al., 2020; Kujawa et al., 2018, 2013; Munsters et al., 2019; Taylor et al., 2016; Webb et al., 2022) only reached fair reliability (*ICC* = 0.52, *n* = 29 measurements, *SE* = 0.04). Pediatric mean amplitude reliability also did not demonstrate a specific trend as time to retest increased (range = 1.5 weeks – 6 years; see Fig. 3D).

**Table 9:** Test-retest reliabilities of adult ERP peak amplitude and mean amplitude studies.

Paper	Age(s) at First Test	Sample Size	Time to Retest	ERP Measure(s)	Reliability (ICC)
Taylor et al. (2016)	19–28 years	N = 32	1–2 weeks	CNV O-wave Component Mean	**0.58**
				CNV E-wave Component Mean	**0.19**
				Total CNV Mean	**0.05**
				N1 Baseline-to-Peak	**0.41**
				N1 Peak-to-Peak	**0.26**
				P2 Baseline-to-Peak	**0.74**
				P2 Peak-to-Peak	**0.71**
				N2 Baseline-to-Peak	**0.65**
				N2 Peak-to-Peak	**0.47**
				P3 Baseline-to-Peak	**0.75**
				P3 Peak-to-Peak	**0.57**
Lin et al. (2020)	18–30 years	N = 53	1–3 weeks	ERN Peak-to-Peak	**0.69**
				Pe Peak-to-Peak	**0.74**
Weinberg and Hajcak (2011)	Mean age of 21.12 years	N = 26	1.5–2.5 years	ERN Peak	**0.62**
				CRN Peak	**0.55**
				ΔERN Peak	**0.60**
				Area Under the ERN	**0.62**
				Area Under the CRN	**0.72**
				Area Difference ΔERN	**0.66**
				Area Under the Pe	**0.68**
				Area Around the ERN Peak	**0.66**
				Area Around the CRN Peak	**0.66**
				Area Around the Δpeak	**0.56**
Levinson et al. (2017)	Undergraduates	N = 59	1 week	RewP Mean	**0.62**
				FN Mean	**0.81**
				ΔRewP Mean	**0.43**
Fallgatter et al. (2001)	22–60 years	N = 23	30 min	P300 Go Peak	**0.85**
				P300 NoGo Peak	**0.92**
Brunner et al. (2013)	Median age 27.5 years	N = 26	6–18 months	P3 NoGo Wave Peak	**0.81**
				IC P3 NoGo Early Peak	**0.85**
				IC P3 NoGo Late Peak	**0.80**
Hämmerer et al. (2012)	Younger adults (mean age = 24.27 years)	N = 47	2 weeks	P2 Peak CPT Go Trials	**0.83**
				N2 Peak CPT Go Trials	**0.79**
				P3 Peak CPT Go Trials	**0.56**
				P2-N2 Peak CPT Go Trials	**0.76**
				P2 Peak Reinforcement Learning Task (Avg Gain/Loss)	**0.59**
				N2 Peak Reinforcement Learning Task (Avg Gain/Loss)	**0.63**
				P3 Peak Reinforcement Learning Task (Avg Gain/Loss)	**0.64**
				P2-N2 Peak Reinforcement Learning Task (Avg Gain/Loss)	**0.67**
	Older adults (mean age = 71.24 years)	N = 47	2 weeks	P2 Peak CPT Go Trials	**0.75**
				N2 Peak CPT Go Trials	**0.76**
				P3 Peak CPT Go Trials	**0.77**
				P2-N2 Peak CPT Go Trials	**0.67**
				P2 Peak Reinforcement Learning Task (Avg Gain/Loss)	**0.83**
				N2 Peak Reinforcement Learning Task (Avg Gain/Loss)	**0.73**
				P3 Peak Reinforcement Learning Task (Avg Gain/Loss)	**0.70**
				P2-N2 Peak Reinforcement Learning Task (Avg Gain/Loss)	**0.78**
Cassidy et al. (2012)	19–35 years old	N = 25	1 month	P1 Peak	**0.76**
				N1 (P08) Peak	**0.87**
				N1 (P07) Peak	**0.91**
				P3a Peak	**0.77**
				P3a Difference Peak	**0.64**
				P3b Peak	**0.77**
				P3b Difference Peak	**0.52**
				ERN Peak	**0.74**
				ERN Difference Peak	**0.87**
				Pe Peak	**0.71**
				Pe Difference Peak	**0.85**
				P400 Peak	**0.85**
				N170 Peak	**0.84**
				ERN Peak-to-Peak	**0.76**
				ERN Difference Peak-to-Peak	**0.56**
				P3a Mean	**0.78**
				P3a Difference Mean	**0.82**
				P3b Mean	**0.80**
				P3b Difference Mean	**0.73**
				Pe Mean	**0.62**
				Pe Difference Mean	**0.74**
				P400 Mean	**0.85**
				Area Under the P3a	**0.78**
				Area Difference P3a	**0.82**
				Area Under the P3b	**0.83**
				Area Difference P3b	**0.59**
				Area Under the Pe	**0.54**
				Area Difference Pe	**0.78**
				Area Under the P400	**0.80**
Huffmeijer et al. (2014)	18–22 years	N = 10	4 weeks	VPP Mean	**0.95**
				N170 Mean (Avg Left/Right)	**0.91**
				MFN Mean	**0.09**
				P3 Mean (Avg Left/Right)	**0.63**
				LPP Mean	**0.85**
Segalowitz et al. (2010)	Mean age of 28.2 years	N = 11	20 min	ERN (Fz) Peak-to-Peak	**0.66**
				ERN (FCz) Peak-to-Peak	**0.79**
				ERN (Cz) Peak-to-Peak	**0.73**
Sinha et al. (1992)	Mean age of 36.48 years	N = 44	14 months	Visual N1 Peak (Avg Oz, Cz, Pz)	**0.69**
				Visual N2 Peak (Avg Oz, Cz, Pz)	**0.66**
				Visual P3 Peak (Avg Cz, Pz)	**0.69**
				Auditory N1 Peak (Cz)	**0.66**
				Auditory N2 Peak (Cz)	**0.47**
				Auditory P3 Peak (Avg Cz, Pz)	**0.56**
Kinoshita et al. (1996)	29–52 years	N = 10	1 week	P300 Baseline-to-Peak	**0.49**
				N100 Baseline-to-Peak	**0.58**
				N200 Baseline-to-Peak	**0.51**
				N100-P300 Peak-to-Peak	**0.48**
				N200-P300 Peak-to-Peak	**0.54**
Rentzsch et al. (2008)	19–51 years	N = 41	4 weeks	P50 Base-to-Peak	**0.86**
				N100 Base-to-Peak	**0.71**
				P200 Base-to-Peak	**0.82**
				P50 Peak-to-Peak	**0.89**
				N100 Peak-to-Peak	**0.70**
				P200 Peak-to-Peak	**0.78**
Malcolm et al. (2019)	Mean age of 24.2 years	N = 12	Mean of 2.3 years	Frontocentral N2 Mean	**0.42**
				Central N2 Mean	**0.61**
				Centroparietal N2 Mean	**0.28**
				Frontocentral P3 Mean	**0.61**
				Central P3 Mean	**0.61**
				Centroparietal P3 Mean	**0.40**
Thesen and Murphy (2002)	Younger adults and elderly	N = 20	4 weeks	N1 Baseline-to-Peak	**0.51**
				P2 Baseline-to-Peak	**0.27**
				P3 Baseline-to-Peak	**0.50**
				N1-P2 Peak-to-Peak	**0.55**
				N1-P3 Peak-to-Peak	**0.52**
D.M. Olvet and Hajack (2009)	Undergraduates	N = 45	2 weeks	CRN Peak	**0.58**
				ERN Peak	**0.70**
				ERN-CRN Difference Peak	**0.51**
				Area Under the CRN	**0.78**
				Area Under the ERN	**0.70**
				Area Difference ERN-CRN	**0.47**
				Area Under the Pe	**0.75**
Larson et al. (2010)	19–29 years	N = 20	2 weeks	ERN Mean	**0.66**
				CRN Mean	**0.75**
				Pe Mean (Error Trials)	**0.48**
				Pe Mean (Correct Trials)	**0.68**
Sandre et al. (2020)	Mean age of 18.2 years	N = 33	5 months	ERN Peak (Cz)	**0.67**
				ERN Peak (FCavg)	**0.57**
				CRN Peak (Cz)	**0.67**
				CRN Peak (FCavg)	**0.66**
				ΔERN Peak (Cz)	**0.47**
				ΔERN Peak (FCavg)	**0.39**
				ERN Peak-to-Peak (Cz)	**0.56**
				ERN Peak-to-Peak (FCavg)	**0.39**
				CRN Peak-to-Peak (Cz)	**0.54**
				CRN Peak-to-Peak (FCavg)	**0.21**
				ΔERN Peak-to-Peak (Cz)	**0.46**
				ΔERN Peak-to-Peak (FCavg)	**0.15**
				ERN Mean (Cz)	**0.62**
				ERN Mean (FCavg)	**0.59**
				CRN Mean (Cz)	**0.71**
				CRN Mean (FCavg)	**0.62**
				ΔERN Mean (Cz)	**0.46**
				ΔERN Mean (FCavg)	**0.26**
Suchan et al. (2018)	20–28 years	N = 14	28 days	ERN Peak (Cz)	**0.89**
				CRN Peak (Cz)	**0.74**
				ERN-CRN Difference Peak (Cz)	**0.63**
				ERN Peak (FCz)	**0.95**
				CRN Peak (FCz)	**0.75**
				ERN-CRN Difference Peak (FCz)	**0.79**
				Area Under the ERN (Cz)	**0.83**
				Area Under the CRN (Cz)	**0.80**
				Area Difference ERN-CRN (Cz)	**0.74**
				Area Under the ERN (FCz)	**0.81**
				Area Under the CRN (FCz)	**0.68**
				Area Difference ERN-CRN (FCz)	**0.59**
Hall et al. (2006)	19–55 years	N = 19	Mean of 17.8 days	MMN Peak	**0.67**
				P300 Peak	**0.86**
				P50 Peak (Conditioning Paradigm)	**0.56**
				P50 Peak (Testing Paradigm)	**0.57**
				MMN Mean	**0.66**
Wang et al. (2021)	18–25 years	N = 16	3–4 days	Duration-Related MMN Peak	**0.70**
				Frequency-Related MMN Peak	**0.73**
Lew et al. (2007)	18–58 years (Healthy Control Group)	N = 21	Median of 6.5 days	N1 Peak	**0.66**
				MMN Peak	**0.60**
				P3 Peak	**0.84**
				N4 Peak	**0.63**
	20–53 years (TBI Group)	N = 7	Median of 6.5 days	N1 Peak	**0.70**
				MMN Peak	**0.21**
				P3 Peak	**-0.02**
				N4 Peak	**0.31**
Light and Braff (2005)	Adults (Schizophrenia Group)	N = 10	Mean of 578 days	MMN Mean	**0.77**
Chen et al. (2018)	20–26 years	N = 20	7 hours	MMN Mean (Happy Silent Movie Task)	**0.41**
				MMN Mean (Happy 2-Back Working Memory Task)	**0.11**
				MMN Mean (Angry Silent Movie Task)	**0.29**
				MMN Mean (Angry 2-Back Working Memory Task)	**0.48**
			2 weeks	MMN Mean (Happy Silent Movie Task)	**0.40**
				MMN Mean (Happy 2-Back Working Memory Task)	**0.54**
				MMN Mean (Angry Silent Movie Task)	**0.49**
				MMN Mean (Angry 2-Back Working Memory Task)	**0.24**
Jiao et al. (2022)	Adults (Schizophrenia Group)	N = 34	2 days	Duration-Related MMN Peak	**> 0.60**
				Frequency-Related MMN Peak	**< 0.40**

**Table 10:** Test-retest reliabilities of pediatric ERP peak amplitude and mean amplitude studies.

Paper	Age(s) at First Test	Sample Size	Time to Retest	ERP Measure(s)	Reliability (ICC)
Munsters et al. (2019)	9–10 months	N = 31	2 weeks	N290 Mean	**0.76**
				P400 Mean	**0.58**
				Nc Mean	**0.57**
Kompatsiari et al. (2016)	Mean age 12.2 years (ADHD Group)	N = 22	30 min	P1 Peak Occipital	**0.96**
				N1 Peak Occipital	**0.86**
				P2 Peak NoGo Wave	**0.94**
				N2 Peak NoGo Wave	**0.68**
				P3 Peak Go Wave	**0.85**
				IC P3 Peak Go Wave	**0.80**
				P3 Peak NoGo Wave	**0.81**
				IC P3 Peak NoGo Wave Early	**0.77**
				IC P3 Peak NoGo Wave Late	**0.78**
Cremone-Caira et al. (2020)	7–11 years (ASD Group)	N = 21 (Flanker)	3 months	N2 Mean 'Congruent' Flanker Task	**0.54**
				N2 Mean 'Incongruent' Flanker Task	**0.63**
		N = 14 (Go/Nogo)	3 months	N2 Mean 'Go' Go/Nogo Task	**0.82**
				N2 Mean 'Nogo' Go/Nogo Task	**0.58**
Taylor et al. (2016)	7–13 years	N = 51	1–2 weeks	CNV E-wave Component Mean	**0.50**
				Total CNV Mean	**0.33**
				N1 Baseline-to-Peak	**0.51**
				N1 Peak-to-Peak	**0.24**
				P2 Baseline-to-Peak	**0.39**
				P2 Peak-to-Peak	**0.53**
				N2 Baseline-to-Peak	**0.53**
				N2 Peak-to-Peak	**0.59**
				P3 Baseline-to-Peak	**0.48**
				P3 Peak-to-Peak	**0.52**
Lin et al. (2020)	8–12 years	N = 118	1–3 weeks	ERN Peak-to-Peak	**0.54**
				Pe Peak-to-Peak	**0.60**
Kujawa et al. (2013)	8–13 years	N = 34	2 years	Neutral Parietal LPP Mean	**0.61**
				Pleasant Parietal LPP Mean	**0.73**
				Unpleasant Parietal LPP Mean	**0.66**
				Neutral Occipital LPP Mean	**0.55**
				Pleasant Occipital LPP Mean	**0.64**
				Unpleasant Occipital LPP Mean	**0.60**
				Pleasant-Neutral Parietal LPP Mean	**0.11**
				Unpleasant-Neutral Partietal LPP Mean	**0.46**
				Pleasant-Neutral Occipital LPP Mean	**0.15**
				Unpleasant-Neutral Occipital LPP Mean	**0.26**
Jetha et al. (2021)	Kindergarten - 1st grade	N = 110	0.8–1.5 years	P1 Peak (O1)	**0.49**
				P1 Peak (O2)	**0.62**
				P1 Peak (Oz)	**0.59**
				N170 Peak (P7)	**0.54**
				N170 Peak (P8)	**0.49**
				VPP Peak (Fz)	**0.48**
				VPP Peak (FC1)	**0.52**
				VPP Peak (FC2)	**0.52**
				VPP Peak (Cz)	**0.62**
Hämmerer et al. (2012)	Children (mean age = 10.15 years)	N = 45	2 weeks	P2 Peak CPT Go Trials	**0.72**
				N2 Peak CPT Go Trials	**0.40**
				P3 Peak CPT Go Trials	**0.61**
				P2-N2 Peak CPT Go Trials	**0.65**
				P2 Peak Reinforcement Learning Task (Avg Gain/Loss)	**0.61**
				N2 Peak Reinforcement Learning Task (Avg Gain/Loss)	**0.44**
				P3 Peak Reinforcement Learning Task (Avg Gain/Loss)	**0.60**
				P2-N2 Peak Reinforcement Learning Task (Avg Gain/Loss)	**0.54**
	Adolescents (mean age = 14.38 years)	N = 46	2 weeks	P2 Peak CPT Go Trials	**0.66**
				N2 Peak CPT Go Trials	**0.59**
				P3 Peak CPT Go Trials	**0.60**
				P2-N2 Peak CPT Go Trials	**0.69**
				P2 Peak Reinforcement Learning Task (Avg Gain/Loss)	**0.77**
				N2 Peak Reinforcement Learning Task (Avg Gain/Loss)	**0.67**
				P3 Peak Reinforcement Learning Task (Avg Gain/Loss)	**0.64**
				P2-N2 Peak Reinforcement Learning Task (Avg Gain/Loss)	**0.66**
Segalowitz et al. (2010)	15 years	N = 28	3–6 weeks	ERN (Fz) Peak-to-Peak Flanker	**0.11**
				ERN (FCz) Peak-to-Peak Flanker	**0.40**
				ERN (Cz) Peak-to-Peak Flanker	**0.59**
				ERN (Fz) Peak-to-Peak Go/NoGo	**0.41**
				ERN (FCz) Peak-to-Peak Go/NoGo	**0.51**
				ERN (Cz) Peak-to-Peak Go/NoGo	**0.61**
Beker et al. (2021)	6–9.4 years (ASD Group)	N = 33	Mean of 5.2 months	VEP N1 Peak	**0.86**
				VEP P1 Peak	**0.79**
				AEP N1 Peak Cue	**0.79**
				AEP P2 Peak Cue	**0.75**
				VEP P2 Peak	**0.72**
				AEP N1 Peak No-Cue	**0.75**
				AEP P1 Peak Cue	**0.44**
				AEP P1 Peak No-Cue	**0.33**
				AEP P2 Peak No-Cue	**0.26**
Kujawa et al. (2018)	9 years	N = 75	3 years	RewP Gain Mean	**0.62**
				RewP Loss Mean	**0.53**
	13 years	N = 75	3 years	RewP Gain Mean	**0.61**
				RewP Loss Mean	**0.57**
	9 years	N = 75	6 years	RewP Gain Mean	**0.51**
				RewP Loss Mean	**0.53**
Webb et al. (2022)	6–11.5 years (TD Group)	N = 119	6 weeks	VEP N1 Peak	**0.68**
				VEP P100 Peak	**0.74**
				P100 Peak Upright Faces	**0.70**
				N170 Peak Upright Faces	**0.71**
				N200 Peak Biological Motion Specificity Effect	**0.10**
				P3 Mean Biological Motion Specificity Effect	**0.15**
				P100 Peak Biological Motion	**0.67**
				N200 Peak Biological Motion	**0.77**
				P3 Mean Biological Motion	**0.71**
	6–11.5 years (ASD Group)	N = 280	6 weeks	VEP N1 Peak	**0.73**
				VEP P100 Peak	**0.70**
				P100 Peak Upright Faces	**0.72**
				N170 Peak Upright Faces	**0.74**
				N200 Peak Biological Motion Specificity Effect	**0.03**
				P3 Mean Biological Motion Specificity Effect	**0.02**
				P100 Peak Biological Motion	**0.67**
				N200 Peak Biological Motion	**0.69**
				P3 Mean Biological Motion	**0.67**

**Table 11:** Test-retest reliabilities of adult ERP latencies (to peak amplitudes) studies.

Paper	Age(s) at First Test	Sample Size	Time to Retest	ERP Measure(s)	Reliability (ICC)
Taylor et al. (2016)	19–28 years	N = 32	1–2 weeks	N1	**0.51**
				P2	**0.59**
				N2	**0.64**
				P3	**0.30**
Lin et al. (2020)	18–30 years	N = 53	1–3 weeks	ERN	**0.33**
				Pe	**0.52**
Weinberg and Hajcak (2011)	Mean age of 21.12 years	N = 26	1.5–2.5 years	ERN	**0.29**
				CRN	**-0.08**
				ΔERN	**-0.14**
Fallgatter et al. (2001)	22–60 years	N = 23	30 min	P300 Go	**0.70**
				P300 NoGo	**0.75**
Brunner et al. (2013)	Median age 27.5 years	N = 26	6–18 months	P3 NoGo Wave Peak	**0.90**
				IC P3 NoGo Early Peak	**0.86**
				IC P3 NoGo Late Peak	**0.79**
Cassidy et al. (2012)	19–35 years old	N = 25	1 month	P1	**0.58**
				N1 (P08)	**0.53**
				N1 (P07)	**0.87**
				P3a	**0.38**
				P3a Difference	**0.88**
				P3b	**0.41**
				P3b Difference	**0.18**
				ERN	**0.45**
				ERN Difference	**0.30**
				Pe	**0.43**
				Pe Difference	**0.39**
				P400	**0.19**
				N170	**0.76**
Huffmeijer et al. (2014)	18–22 years	N = 10	4 weeks	VPP	**0.51**
				N170 (Avg Left/Right)	**0.49**
				MFN	**0.66**
				P3 (Avg Left/Right)	**0.32**
Sinha et al. (1992)	Mean age of 36.48	N = 44	14 months	Visual N1 (Avg Oz, Cz, Pz)	**0.56**
				Visual N2 (Avg Oz, Cz, Pz)	**0.40**
				Visual P3 (Avg Cz, Pz)	**0.24**
				Auditory N1 (Cz)	**0.73**
				Auditory N2 (Cz)	**0.54**
				Auditory P3 (Avg Cz, Pz)	**0.23**
Kinoshita et al. (1996)	29–52 years	N = 10	1 week	P300	**0.38**
				N100	**0.53**
				N200	**0.22**
Rentzsch et al. (2008)	19–51 years	N = 41	4 weeks	P50	**0.73**
				N100	**0.54**
				P200	**0.55**
Malcolm et al. (2019)	Mean age of 24.2 years	N = 12	Mean of 2.3 years	Frontocentral N2	**0.68**
				Central N2	**0.69**
				Centroparietal N2	**0.68**
				Frontocentral P3	**0.51**
				Central P3	**0.55**
				Centroparietal P3	**0.68**
Thesen and Murphy (2002)	Younger adults and elderly	N = 20	4 weeks	N1	**0.50**
				P2	**0.82**
				P3	**0.73**
D.M. Olvet and Hajack (2009)	Undergraduates	N = 45	2 weeks	CRN	**-0.02**
				ERN	**0.42**
				ERN-CRN Difference	**0.24**
Larson et al. (2010)	19–29 years	N = 20	2 weeks	ERN	**0.33**
				CRN	**0.63**
Suchan et al. (2018)	20–28 years	N = 14	28 days	ERN (Cz)	**0.57**
				CRN (Cz)	**0.57**
				ERN-CRN Difference (Cz)	**0.53**
				ERN (FCz)	**0.35**
				CRN (FCz)	**0.14**
				ERN-CRN Difference (FCz)	**0.69**
Hall et al. (2006)	19–55 years	N = 19	Mean of 17.8 days	MMN	**0.34**
				P300	**0.88**
Lew et al. (2007)	18–58 years (Healthy Control Group)	N = 21	Median of 6.5 days	N1	**0.61**
				MMN	**0.70**
				P3	**0.64**
				N4	**-0.08**
	20–53 years (TBI Group)	N = 7	Median of 6.5 days	N1	**0.80**
				MMN	**0.50**
				P3	**-0.17**
				N4	**-0.75**
Chen et al. (2018)	20–26 years	N = 20	7 hours	MMN (Happy Silent Movie Task)	**0.51**
				MMN (Happy 2-Back Working Memory Task)	**0.54**
				MMN (Angry Silent Movie Task)	**0.67**
				MMN (Angry 2-Back Working Memory Task)	**0.68**
			2 weeks	MMN (Happy Silent Movie Task)	**0.37**
				MMN (Happy 2-Back Working Memory Task)	**0.41**
				MMN (Angry Silent Movie Task)	**0.65**
				MMN (Angry 2-Back Working Memory Task)	**0.61**
Jiao et al. (2022)	Adults (Schizophrenia Group)	N = 34	2 days	Duration-Related MMN	**0.34**
				Frequency-Related MMN	**0.02**

**Table 12:** Test-retest reliabilities of pediatric ERP latencies (to peak amplitudes) studies.

Paper	Age(s) at First Test	Sample Size	Time to Retest	ERP Measure(s)	Reliability (ICC)
Kompatsiari et al. (2016)	Mean age 12.2 years (ADHD Group)	N = 22	30 min	P1 Occipital	**0.89**
				N1 Occipital	**0.75**
				P2 NoGo Wave	**0.90**
				N2 NoGo Wave	**0.70**
				P3 Go Wave	**0.31**
				IC P3 Go Wave	**0.72**
				P3 NoGo Wave	**0.69**
				IC P3 NoGo Wave Early	**0.83**
				IC P3 NoGo Wave Late	**0.78**
Taylor et al. (2016)	7–13 years	N = 51	1–2 weeks	N1	**0.21**
				P2	**0.31**
				N2	**0.21**
				P3	**0.29**
Lin et al. (2020)	8–12 years	N = 118	1–3 weeks	ERN	**0.16**
				Pe	**0.18**
Jetha et al. (2021)	Kindergarten - 1st grade	N = 110	0.8–1.5 years	P1 (O1)	**0.47**
				P1 (O2)	**0.30**
				P1 (Oz)	**0.30**
				N170 (P7)	**0.49**
				N170 (P8)	**0.60**
				VPP (Fz)	**0.52**
				VPP (FC1)	**0.54**
				VPP (FC2)	**0.45**
				VPP (Cz)	**0.48**
Webb et al. (2022)	6–11.5 years (TD Group)	N = 119	6 weeks	VEP P100	**0.59**
				P100 Upright Faces	**0.69**
				N170 Upright Faces	**0.75**
	6–11.5 years (ASD Group)	N = 280	6 weeks	VEP P100	**0.70**
				P100 Upright Faces	**0.68**
				N170 Upright Faces	**0.66**

As with internal consistency, the test-retest reliability of ERP latency measurements was lower than for ERP peak amplitude and mean amplitude measurements in adults. Across the adult studies (Brunner et al., 2013; Cassidy et al., 2012; Chen et al., 2018; Fallgatter et al., 2001; Hall et al., 2006; Huffmeijer et al., 2014; Jiao et al., 2022; Kinoshita et al., 1996; Larson et al., 2010; Lew et al., 2007; Lin et al., 2020; Malcolm et al., 2019; Olvet and Hajcak, 2009a; Rentzsch et al., 2008; Sinha et al., 1992; Suchan et al., 2019; Taylor et al., 2016; Thesen and Murphy, 2002; Weinberg and Hajcak, 2011), latency test-retest reliability was generally only fair (*ICC* = 0.48, *n* = 83 measurements, *SE* = 0.03) regardless of time to retest (range = 30 min – 2.3 years, see Fig. 3E). The average ERP latency reliability in pediatric populations was also only fair (*ICC* = 0.54, *n* = 30 measurements, *SE* = 0.04; Jetha et al., 2021; Kompatsiari et al., 2016; Lin et al., 2020; Taylor et al., 2016; Webb et al., 2022), although slightly higher than ERP latency reliability in adult studies. There was no discernable trend as time to retest increased in the pediatric reliability papers, and we draw no conclusions from this pattern given the limited number of studies involved (see Fig. 3F).

#### 3.2.3 ERP measurement recommendations

To summarize, the reliability profile of ERP measurements in extant studies depends on both the type of ERP measure (peak amplitude vs. mean amplitude vs. latency) and the population that is being studied (adult vs. pediatric). ERP peak amplitude and mean amplitude are far more consistent than ERP latency to peak amplitude in the papers reviewed here across the lifespan. Further, while ERP peak and mean amplitudes often achieved similar internal consistency levels in adults, ERP mean amplitude achieved far better levels of internal consistency than peak amplitude in pediatric samples, elevating internal consistency estimates in pediatric samples to the same level as seen in adult studies. We note that Luck et al. (2021) have recently raised important issues with respect to measuring internal consistency for peak (amplitude and latency) ERP measures, as the average ERP waveform does not have the same peak properties as the average of the individual trial values. Researchers can shift to bootstrapped estimates of internal consistency for such peak measures moving forward (Boudewyn et al., 2018; Luck et al., 2021) (and indeed all software recommended for calculating internal consistency in this manuscript uses bootstrapped estimates of internal consistency). Test-retest reliability for ERP measures was highly variable at all ages and requires further examination to optimize these measures for most individual difference analyses.

Given current evidence, we recommend the following: 1. In general, great care is needed when measuring ERP latencies to peak amplitude, and further studies should seek to optimize these measurements across the lifespan, as well as the measurement of their internal consistency through bootstrapped methods (see Luck et al., 2021). Still, evidence to date suggests this type of measurement makes for poor candidate individual difference markers across the lifespan. In addition to the need for bootstrapped approaches to adequately measure internal consistency for peak measures like latency to peak amplitude, as speculated by Olvet and Hajcak (2009a) and Weinberg and Hajcak (2011), reliability may be poor for ERP latencies due to high variability in response times on the individual level. There may also be error contributed by changing or imperfect equipment's stimulus presentation timing precision over the course of the experiment that increases error in latency measurements relative to amplitude measurement. 2. Though ERP peak amplitudes generally provide adequate reliability, they may not reflect the optimal way to index ERPs for individual differences analyses, especially for pediatric samples. That is, mean amplitude for a given component has been shown to be a more robust ERP measure in these regards (Clayson et al., 2013), and we encourage researchers to consider this alternative measurement moving forward (to that end, our open-source software for calculating ERPs, HAPPE+ER (Monachino et al., 2022), provides mean amplitudes as standard calculated measures in the GenerateERPs function). 3. We note that the papers included here mainly used cognitive tasks to measure ERPs, so less is known about how reliable these measurements are across a wider range of paradigms (e.g., perceptual or affective tasks). Future research should assess the generalization of these reliability patterns. 4. Importantly, there is also a startling lack of work evaluating ERP internal consistency or test-retest reliability in infancy, with only one study found at the time of this review (Munsters et al., 2019). It is critical that the field conducts reliability assessments before middle childhood.

### 3.3 Nonlinear measures

A rapidly growing literature quantifies EEG signal dynamics by measuring nonlinear time series characteristics. Though originally applied to EEG data in 1985 (Babloyantz et al., 1985), nonlinear measures have only recently begun to gain traction in EEG analyses, in part because technological advances can now more readily handle their computational burden. These nonlinear measurements most commonly capture the variability and/or predictability of the EEG signal across different timescales and/or frequencies (Stam, 2005). Importantly, there is not yet consensus about their optimal measurement parameters, including the segment lengths over which they are calculated, which impacts the reliability of their measurement. It is also important to note that some measures, like entropy, reflect different dynamics over short vs. long timescales (i.e., local circuit dynamics over short segment length vs. large-scale/long-range dynamics over longer segment lengths). Given the lack of methodological consensus for extracting these features, below we provide the parameterization details for each study to guide interpretation and parameter choices for future studies based on these reliability results. A variety of nonlinear measures have recently been used in efforts to predict autism spectrum disorder outcomes (Bosl et al., 2011, 2018; Peck et al., 2021), to account for individual differences in infant social behavior (Puglia et al., 2020), and to track changes in brain dynamics with age or with different clinical populations (Catarino et al., 2011; Namazi and Jafari, 2019; van Noordt and Willoughby, 2021; Zhang et al., 2009). Though there is burgeoning interest in the functional significance of these features, there is unfortunately very little literature on their consistency, optimization, or reliability.

#### 3.3.1 Reliability of nonlinear measures

**Fig. 4:**
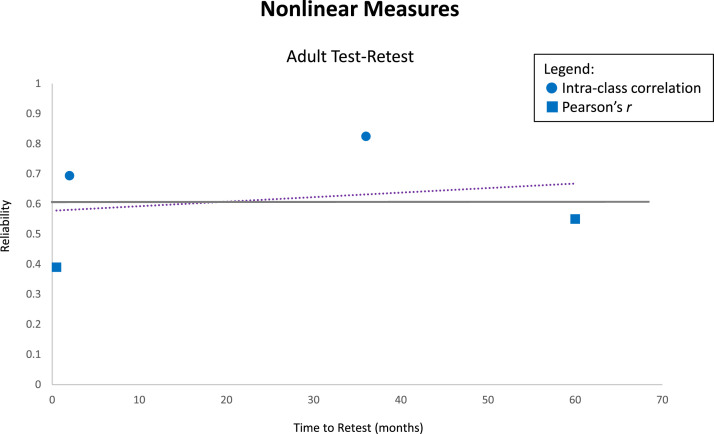
Test-retest values calculated using both intra-class correlations and Pearson's *r* for each adult nonlinear measure study based on time between testing sessions (in months).

The limited research to date suggests nonlinear features may be promising candidates for individual difference analyses. To the best of our knowledge, there is only one assessment of the internal consistency of nonlinear measures to date. Kuntzelman et al. (2018) found all internal consistency values to be > 0.80 for a number of entropy metrics evaluated across the whole scalp for the total EEG signal (split into eight 30 second segments to compute Cronbach's alpha). Moreover, only a few studies have assessed the test-retest reliability of nonlinear baseline EEG measures across time, all in adult populations (see Table 13). First, Kuntzelman et al. (2018) found good-excellent test-retest reliability over a week for multiple entropy measures taken on very short timescales (up to 100 milliseconds) for most scalp locations and frequency bands (though noted lower occipital reliability, lower reliability for permutation entropy, and sometimes lower delta and gamma band reliability). Similarly, Gudmundsson et al. (2007) report good whole-brain test-retest reliability (*ICC* = 0.69) of Hjorth parameters, sample entropy, singular value decomposition (SVD) entropy, and permutation entropy when considering measure averages from 5-second segments with 50% overlapping windows. Second, Põld et al. (2021) report excellent whole- brain test-retest reliability (*ICC* = 0.83) of the Higuchi fractal dimension and detrended fluctuation analysis based on the median value from 20.48-second EEG segments. However, Dünki et al. (2000) found only fair correlations over 14 days (r = 0.59) and 5 years (r = 0.39) for the Grassberger-Procaccia correlation dimension in adults. Together these studies indicate at least some nonlinear measures demonstrate adequate reliability in adult populations and merit further exploration (Fig. 4).

**Table 13:** Test-retest reliabilities of adult nonlinear measures studies.

Paper	Age(s) at First Test	Sample Size	Time to Retest	Reliability Measure(s)	Nonlinear Measure(s)	Reliability (ICC)
Gudmundssen et al. (2007)	Mean age of 71.7 years	N = 15	2 months	ICC	Hjorth: Activity	**0.76**
					Hjorth: Mobility	**0.66**
					Hjorth: Complexity	**0.68**
					Sample entropy	**0.69**
					SVD entropy	**0.71**
					Permutation entropy	**0.66**
					Lempel-Ziv Complexity	**0.70**
Pold et al. (2020)	Mean age of 42.3 years	N = 17	3 years	ICC	Higuchi Fractal Dimension	**0.81**
					Detrended Flucation Analysis	**0.84**
Dunki et al. (2000)	Mean age of 28 years	N = 30	14 days	Pearson's *r*	Correlation dimension	**0.39**
			5 years		Correlation dimension	**0.55**

#### 3.3.2 Nonlinear measurement recommendations

Broadly speaking, there is a pressing need to optimize and assess both the internal consistency and test- retest reliability of nonlinear time series measures across the lifespan given that the application of these methodologies is relatively new for the field. Early investigations show great promise for this class of measures that facilitate characterizing the nonlinear dynamics of the brain, a fundamentally nonlinear system. Understanding the parameter settings that produce the most reliable estimates of these nonlinear dynamics can lead to standardized methodology for extracting nonlinear features for individual difference analyses. Further, to our knowledge no one has yet reported nonlinear measure internal or test-retest reliability in pediatric populations, which must change before these features are used further in individual difference analyses.

### 3.4 Functional connectivity measures

Functional connectivity measures in EEG data examine the relation of brain activity across different physical regions of the brain or scalp to characterize neural network function.

Functional connectivity can be measured both in source space (the areas of the brain from which the signals originate) and scalp space (the areas on the scalp where the signal is detected by the EEG sensors), and may include all frequencies within the EEG signal or calculations within specific frequency bands (e.g., delta, alpha). Common approaches to measure functional connectivity include both amplitude and phase-based methods, like amplitude envelope coupling (Bruns et al., 2000), phase lag index (PLI; Stam, 2005), and variants (e.g., debiased weighted phase lag index), to study synchronization between sensors/brain regions. Methods like graph theoretical methods can extract additional features to describe both local and global scalp/brain network characteristics using the functional connectivity estimates (e.g., average clustering coefficient, path length, and the small-world index (SWI), etc.). Such EEG measures have been used in the context of understanding language (Gaudet et al., 2020), dyslexia (Dushanova et al., 2020), epilepsy (Sargolzaei et al., 2015), autism (Catarino et al., 2013; Haartsen et al., 2019; O'Reilly et al., 2017; Righi et al., 2014), and physical development (Grieve et al., 2008; Xie et al., 2019). Unfortunately, and likely due to the recency of their adoption, there is very little existing literature that examines the reliability of functional connectivity measures, especially in studies with pediatric samples.

#### 3.4.1 Internal consistency of functional connectivity measures

**Fig. 5:**
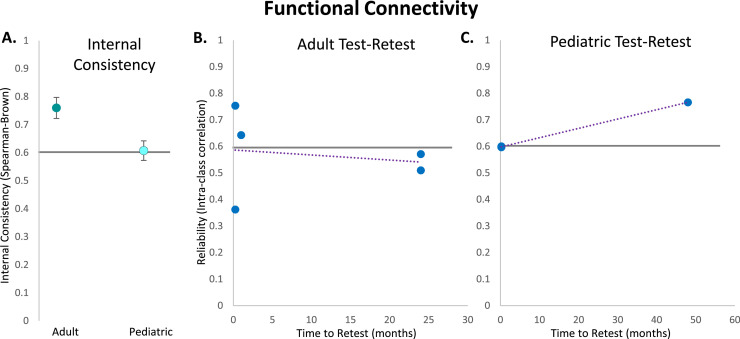
Internal consistency and test-retest reliability of functional connectivity measures. A: Average internal consistency values calculated using the Spearman-Brown Formula for all adult and pediatric studies. B: Test-retest values calculated using intra-class correlations for each adult functional connectivity study based on time between testing sessions (in months). C: Test-retest values calculated using intra-class correlations for each pediatric connectivity study based on time between testing sessions (in months).

To our current knowledge, there are only two studies examining the internal consistency of functional connectivity measures at any age, both within task contexts (Miskovic and Keil, 2015; Morales et al., 2022; see Tables 14 and 15). In adults, Miskovic and Keil (2015) examined coherence and phase synchrony in a visual-evoked potential task paradigm, while Morales et al. (2022) examined phase synchrony in delta and theta bands in children participating in a cognitive Go/No-Go paradigm. The adult functional connectivity measures demonstrated excellent internal consistency (ρ *= 0*.76, *n* = 2 measurements, *SE* = 0.04) across all measures using Spearman rank order correlations (Miskovic and Keil, 2015), while in childhood the measures produced good internal consistency (*rSB = 0*.60, *n* = 2 measurements, *SE* = 0.04) using Spearman Brown methods (Morales et al., 2022; Fig. 5A). However, there are too few studies to draw conclusions yet about what measurement parameters and kinds of functional connectivity measures provide adequate internal consistency for individual difference analyses.

**Table 14:** Internal consistency of adult functional connectivity studies.

Paper	Age(s)	Sample Size	Paradigm	Consistency Measure	Functional Connectivity Measure(s)	Frequency Band	Consistency
Miskovic and Keil (2014)	Mean age 19.14 years	N = 14	Steady-state VEP	Spearman Rho	Coherence	14 Hz	**0.80**
					Phase Synchrony	14 Hz	**0.73**

**Table 15:** Internal consistency of pediatric functional connectivity studies.

Paper	Age(s)	Sample Size	Paradigm	Consistency Measure	Functional Connectivity Measure(s)	Frequency Band	Consistency
Morales et al. (2022)	4–9 years	N = 326	Task-related (Go/No-Go)	Spearman-Brown	Phase Synchrony	Delta	**0.64**
					Phase Synchrony	Theta	**0.57**

#### 3.4.2 Test-retest reliability of functional connectivity measures

Test-retest reliability of EEG functional connectivity measures has been investigated more often than internal consistency, largely in baseline EEG data (Büchel et al., 2021; Cannon et al., 2012; Haartsen et al., 2019; Hardmeier et al., 2014; Hatz et al., 2016; Knyazev et al., 2019; Kuntzelman and Miskovic, 2017; Velde et al., 2019; see Tables 16 and 17). Looking across connectivity measures in adult studies, these features broadly have shown inadequate test-retest reliability to date, demonstrating only fair reliability across the available evidence *(ICC = 0*.56, *n* = 52 measurements, *SE* = 0.03; Büchel et al., 2021; Cannon et al., 2012; Hardmeier et al., 2014; Hatz et al., 2016; Kuntzelman and Miskovic, 2017; Fig. 5B). However, there also appear to be notable differences in measured reliability between the most commonly assessed functional connectivity measures in adult studies, coherence and phase lag index (PLI). Available evidence suggests that coherence demonstrates good reliability while phase lag index only exhibits fair reliability (coherence: *ICC = 0*.67, *n* = 14 measurements, *SE* = 0.08; PLI: *ICC = 0*.54, *n* = 17 measurements, *SE* = 0.05; Büchel et al., 2021; Cannon et al., 2012; Hardmeier et al., 2014; Kuntzelman and Miskovic, 2017). Several papers have examined reliability in pediatric populations and find that across measures, overall reliability is considered good (*ICC = 0*.62, *n* = 29 measurements, *SE* = 0.04; Haartsen et al., 2019; Knyazev et al., 2019; Velde et al., 2019; Fig. 5C). Unfortunately, in this sample of seven papers, there is very little overlap in the exact functional connectivity measures included or in the frequency bands examined. Thus, there is insufficient evidence to date to draw conclusions about how to optimize functional connectivity measurements for individual difference analyses.

**Table 16:** Test-retest reliability of adult functional connectivity studies.

Paper	Age(s) at First Test	Sample Size	Paradigm	Time to Retest	Functional Connectivity Measure(s)	Frequency Band	Reliability (ICC)
Cannon et al. (2012)	Mean age of 20.7 years	N = 19	Resting-state	30 days	Coherence	Delta	**0.88**
						Theta	**0.91**
						Alpha	**0.93**
						Beta	**0.94**
					Phase Lag Index	Delta	**0.09**
						Theta	**0.35**
						Alpha	**0.57**
						Beta	**0.49**
Hardmeier et al. (2014)	20–49.5 years	N = 35	Resting-state	follow up at 1 and 2 years	Phase Lag Index	Theta	**0.75**
						Alpha	**0.77**
						Beta	**0.69**
					Clustering Coefficient	Theta	**0.61**
						Alpha	**0.51**
						Beta	**0.56**
					Path Length	Theta	**0.60**
						Alpha	**0.42**
						Beta	**0.45**
					Small-World Index	Theta	**0.53**
						Alpha	**0.45**
						Beta	**0.54**
Hatz et al. (2016)	20–68 years	N = 40	Resting-state	follow up at 1 and 2 years	Clustering Coefficient	Theta	**0.80**
						Alpha	**0.70**
						Beta	**0.44**
					Path Length	Theta	**0.62**
						Alpha	**0.66**
						Beta	**0.43**
					Degree Correlation	Theta	**0.20**
						Alpha	**0.21**
						Beta	**0.07**
					Degree Diversity	Theta	**0.79**
						Alpha	**0.76**
						Beta	**0.42**
Kuntzelman and Miskovic (2017)	18–22 years	N = 15	Resting-state	1 week	Coherence	Delta	**0.51**
						Theta	**0.66**
						Alpha	**0.30**
						Beta	**0.09**
						Gamma	**0.02**
					Phase Lag Index	Delta	**0.18**
						Theta	**0.47**
						Alpha	**0.59**
						Beta	**0.59**
						Gamma	**0.21**
Büchel et al. (2021)	Mean age of 24.5 years	N = 15	Resting-state	1 week	Weighted Phase Lag Index	Theta	**0.52**
						Alpha-1 (8–10.5 Hz)	**0.90**
						Alpha-2 (10.5–13 Hz)	**0.73**
						Beta-1 (13–20 Hz)	**0.72**
						Beta-2 (20–30 Hz)	**0.52**
					Coherence	Theta	**0.73**
						Alpha-1 (8–10.5 Hz)	**0.88**
						Alpha-2 (10.5–13 Hz)	**0.87**
						Beta-1 (13–20 Hz)	**0.84**
						Beta-2 (20–30 Hz)	**0.82**

**Table 17:** Test-retest reliability of pediatric functional connectivity studies.

Paper	Age at First Test	Sample Size	Paradigm	Time to Retest	Functional Connectivity Measure(s)	Frequency Band	Reliability (ICC)
van der Velde et al. (2019)	10 months	N = 60	Task-related	1 week	Phase Lag Index	Delta	**0.16**
						Theta	**0.87**
						Alpha	**0.84**
						Beta	**0.73**
						Gamma	**0.55**
					Clustering Coefficient	Delta	**0.59**
						Theta	**0.91**
						Alpha	**0.86**
						Beta	**0.73**
						Gamma	**0.62**
					Path Length	Delta	**0.53**
						Theta	**0.89**
						Alpha	**0.84**
						Beta	**0.72**
						Gamma	**0.59**
					Small-World Index	Delta	**0.25**
						Theta	**0.56**
						Alpha	**0.44**
						Beta	**0.14**
						Gamma	**0.13**
Knyazev et al. (2019)	6–11.5 years	N = 68	Resting-state	4 consecutive years (4 waves)	Slow-Fast Wave Coupling	Delta-Alpha	**0.76**
						Delta-Beta	**0.75**
						Theta-Alpha	**0.75**
						Theta-Beta	**0.78**
						N/A	**0.79**
Haartsen et al. (2020)	10 month olds	N = 64	Task-related	1 week	Phase Lag Index	Alpha	**0.86**
					Clustering Coefficient	Alpha	**0.57**
					Path Length	Alpha	**0.44**
					Small-World Index	Alpha	**0.40**

#### 3.4.3 Functional connectivity measure recommendations

Though early studies indicate some functional connectivity approaches may be promising candidates for individual difference analyses, we must further assess reliability for functional connectivity measurements across the lifespan. There remain a variety of functional connectivity measures without any test of internal consistency or test-retest reliability at any age. Furthermore, both studies testing internal consistency of functional connectivity features have examined task paradigms, so studies should look at the internal consistency of resting-state functional connectivity measurements as well.

#### 3.4.4 Individual actions to implement reliability recommendations

Looking across the internal consistency and test-retest reliability results of the EEG measures summarized above, we offer several actions that individual researchers can perform to improve current research practices for individual difference analyses. We also note that this empirical information is complementary to other factors, including topical theory, in research design (e.g., which ages, time-windows between testing, measures of interest). Researchers may integrate this information about reliability of EEG measures along with theoretical and practical motivations and constraints to ultimately inform their design and analyses. We make the following recommendations:

1) Calculate internal consistency values for each study measure in individual-difference analyses. Given that reliability is a property of the measurement (including hardware, acquisition settings, pre-processing, extraction parameters and methods) which continues to vary across sites and studies, routine internal consistency assessments are necessary for researchers seeking to conduct individual difference analyses for a project. Fortunately, if a researcher can extract multiple measurements of the feature(s) of interest (e.g., multiple trials of task-related functional connectivity), they have the data to report internal consistency for measures in studies examining individual differences moving forward. We join others (e.g., Parsons et al., 2019) in making this call to action, and we recommend using any of the following freely-available packages to do so. The freely-available R-based package ‘splithalf’ includes a variety of tools, including multiverse reliability assessments to assist with these calculations (Parsons, 2021). Multiverse assessments evaluate how changing multiple parameters within the pre-processing and feature parameterization process each influence feature reliability, and they may be especially useful when optimizing pipelines and features. Our own MATLAB-based software, HAPPE (Gabard-Durnam et al., 2018; Lopez et al., 2022; Monachino et al., 2022), includes scripts that facilitate bootstrapped split-half internal consistency measurements within the HAPPE pipeline flow. Finally, the excellent ERP Reliability Analysis (ERA) Toolbox from Peter Clayson and colleagues (Carbine et al., 2021; Clayson et al., 2021b; Clayson and Miller, 2017a) is a MATLAB-based software that uses generalizability theory to assess internal consistency and estimate test-retest reliability for EEG measures (including non-ERP EEG measures despite the software name). Researchers may choose between these options depending on code fluency, study need, and these differences in functionality.
2) Evaluate test-retest reliability of EEG measures. Assessments of test-retest reliability (both in the short- and long-term) for measures in infancy and early childhood form a particularly stark gap for the field that individuals must step up to address or restrict the types of individual difference analyses performed with these ages. This is especially problematic given the great interest in these measures as potential early biomarkers for developmental disorders and emerging individual behavioral phenotypes. Though challenging to balance the temporal profiles of measurement reliability against the pace of developmental change in early life, it is critical that we prioritize addressing this gap in the field's knowledge in order to explore these types of individuating analyses before middle childhood. Though not designed for this purpose, reporting test-retest reliability metrics for extant longitudinal study designs can help populate this knowledge gap without undue burden on the field. Individuals may return to their longitudinal studies to conduct test-retest analyses or make data publicly-available to support others’ efforts. Understanding the timescale of reliability and developmental change through these longitudinal studies can also inform subsequent study designs targeting test-retest reliability explicitly.
3) Calculate and report trial minimums to achieve the level of internal consistency used in individual difference analyses. Differences in internal consistency and reliability were observed when comparing adult literature with pediatric literature for several measures. However, it is presently unclear if those differences may be due to developmental variability or to differences in task design or data retention across ages. Reporting trial thresholds will help the field disentangle these potential explanations and then optimize data collection and analysis moving forward (Boudewyn et al., 2018). Prior literature has demonstrated that internal consistency and test-retest reliability estimates are affected by the number of trials used to calculate the EEG measure (e.g., Boudewyn et al., 2018; Fischer et al., 2017; Huffmeijer et al., 2014; Larson et al., 2010; Meyer et al., 2013; Olvet and Hajcak, 2009b; Pontifex et al., 2010). For example, Meyer et al. (2013) found differences in the number of trials needed for the ERN to reach good internal consistency based on the task used, with Flanker and Go/No-Go tasks necessitating at least 10 error trials but the Stroop task requiring over 20 error trials. Pediatric and clinical populations often require briefer paradigms than healthy adult populations, leaving fewer trials to attain adequate consistency. EEG from these populations also frequently exhibit greater levels of artifact, which can result in fewer usable trials retained for analysis. The internal consistency calculating software described above in action item 1 can provide this trial threshold information (e.g., 17 trials per person is required to achieve Chronbach's alpha of 0.75 for this sample and EEG measure). Moreover, such testing would help inform future robust study design and analysis (though as Boudewyn et al. (2018) note, there are multiple other factors that must be considered to determine the number of trials to sufficiently power EEG designs, including sample size, effect magnitude, anticipated noise level in the signal, and these factors’ interactions). Individual researchers should calculate trial numbers required for reliable estimates in their sample, remove participants with insufficient trials, and report the retention minimum trial number in manuscripts. In addition to these steps, the following section details another approach individuals can take to positively impact trial retention and reliability assessments across the lifespan.

## 4 Standardized, automated EEG pre-processing practices for individual difference analyses

Robustly-pre-processed EEG data is a critical prerequisite for extracting reliable EEG measures for individual difference analyses. Artifact signal amplitudes can be orders of magnitude larger than signals of neural origin, so they can dramatically skew EEG measure estimates for an individual. Thus, there is a real risk in reporting individual differences that reflect degree of artifact contamination across the sample instead of differences in neural phenomena if pre-processing does not effectively parse artifact from neural data. Until recently, the gold standard for denoising EEG data for analysis involved removing artifact-laden timepoints through subjective manual-editing (i.e., detecting and removing artifacts by visual inspection). Indeed, the overwhelming majority of internal consistency and test-retest results reported above have been generated with manual editing practices. However, the manual-editing approach has multiple disadvantages with respect to individual difference analyses. First, because entire time segments are removed if any channels of interest are determined to have artifact, the process often results in significant data loss for each EEG file (especially for high-density EEG files with many potentially-contaminated channels). This data loss can contribute to less reliable estimates of an individual's EEG measure, especially in pediatric and clinical samples with limited data collected. Second, the subjective nature of manual-editing leads to variance both between and within scientists with respect to how artifact in data is handled. In the case where “double coding” editing decisions are used as an attempt to mitigate the effects of variance between individuals, reliability between coders is rarely reported. Given that inter-rater reliability is one of the few quantifiable aspects of manual artifact removal addressing the issue of subjectivity, it is helpful that those who do continue to use manual editing at least report this metric. However, reporting inter-rater reliability does not completely remove the subjectivity of manual-editing and still does not offer any quantifiable information about the quality of the data retained. Third, manual-editing is time-intensive and extremely difficult to scale as sample sizes increase to better power individual difference analyses. Below we discuss two alternative strategies to improve individual differences analyses with EEG measures by using: 1) standardized, automated pre-processing pipelines for EEG denoising, and 2) empirical measures of data quality. We then provide recommendations for implementing these strategies moving forward.

### 4.1 Automated EEG denoising

The first strategy to address the setbacks of manual editing and facilitate more robust individual difference analyses across the lifespan is the use of standardized, automated pre-processing software to reduce and remove artifacts. This is a nascent but rapidly growing focus for EEG research (Dien, 2010; Gabard-Durnam et al., 2018; Haresign et al., 2021; Lawhern et al., 2013; Leach et al., 2020; Lopez et al., 2022; Mognon et al., 2011; Monachino et al., 2022; Nolan et al., 2010; Ouyang et al., 2022; Winkler et al., 2011). With the breadth of available tools comes a range of approaches to EEG data pre-processing, spanning fully-automated pipelines, individual scripts, and toolboxes built to aid in different stages (Andersen, 2018; PREP, Bigdely-Shamlo et al., 2015; Cassani et al., 2017; SASICA, Chaumon et al., 2015; APP, da Cruz et al., 2018; MADE, Debnath et al., 2020; EEG-IP-L, Desjardins et al., 2021; ERP PCA Toolkit, Dien, 2010; APICE, Fló et al., 2022; HAPPE 1.0, Gabard-Durnam et al., 2018; MNE, Gramfort et al., 2014; Hatz et al., 2015; Adjusted- ADJUST, Leach et al., 2020; HAPPILEE, Lopez et al., 2022; HAPPE+ER, Monachino et al., 2022; ASR, Mullen et al., 2013; FASTER, Nolan et al., 2010; FieldTrip, Oostenveld et al., 2011; Automagic, Pedroni et al., 2019; APPLESEED, Puglia et al., 2021; EPOS, Rodrigues et al., 2021; Brainstorm, Tadel et al., 2011; miniMADE, Troller-Renfree et al., 2021; MARA, Winkler et al., 2014). Importantly, many of these software implement automated artifact correction approaches that do not sacrifice timepoints, like wavelet-thresholding and independent component analysis, that outperform manual-editing (either timepoint removal or independent component rejection) in successful artifact removal and in the degree of data retained (e.g., MADE, Debnath et al., 2020; HAPPE 1.0, Gabard-Durnam et al., 2018; HAPPILEE, Lopez et al., 2021; HAPPE+ER, Monachino et al., 2021; MARA, Winkler et al., 2014). Thus, standardized, automated pipelines are not only more efficient and consistent in their treatment of artifact across individuals, they may also improve data quality and increase analysis power through both greater participant and data retention rates. There are no issues in scaling this software for large datasets either. The standardized, automated software approach for EEG denoising can in principle address all three primary concerns about manual editing practices with respect to individual difference analyses. However, as with any tool, they may also be used inappropriately and lead to poor performance if care isn't taken in software selection.

While having a broad range of automated EEG software to pick from can seem daunting, each software solution is validated for use in a limited set of contexts, so several factors may guide user choice. First, what kind of populations are involved? The vast majority of existing software options are validated using data only from healthy adults, while few others are validated only with healthy pediatric samples at one or two ages (though see HAPPE+ER (Monachino et al., 2022), which has been validated on adult and pediatric data). Unfortunately, software that is only validated on adult data is often not generalizable to the nature of data and artifacts from pediatric EEG studies. Researchers should select software validated for the same ages or at least the same part of the lifespan as their sample. Second, how many channels of EEG data were collected? The majority of software are only compatible and validated with high-density channel layouts, and often use pre-processing approaches like independent component analysis that are not suitable for some lower-density layouts (though see Cassani et al., 2017; Hajra et al., 2020; HAPPILEE, Lopez et al., 2022; miniMADE, Troller-Renfree et al., 2021). To better support comparisons across studies from a lifespan perspective, concerted effort is needed to develop and validate software across multiple ages, populations, and acquisition setups to overcome these barriers.

If multiple standardized pipelines have been validated for the population(s) and types of EEG data at hand, how might they be compared to guide choice? Researchers have few empirical or conceptual comparisons in the literature currently to inform decisions (but see a recent discussion by Buzzell et al. (2023) on a subset of available pipeline options). Unfortunately, pipelines also vary in the output metrics they provide about what changes have occurred to the data during denoising. This makes it difficult for individual researchers to compare performance across software with such metrics. Publicly-available EEG datasets that could be used across software validation efforts would facilitate comparisons, but there is a dearth of such data from prior software development (note, to this end, we and others have begun releasing validation datasets: Levin et al., 2017; Lopez et al., 2021; Monachino et al., 2021). Researchers may compare software performance on their own sample through visualizations and empirical comparisons. Automated pre-processing does not exempt any researcher from examining and understanding their data. We also recommend researchers use of ground-truth signals (e.g., simulated EEG signals) for comparing software performance. For example, to facilitate such software comparisons more widely, HAPPE software now includes realistic simulated ERP signals (e.g., visual evoked potential, oddball P3 potential, etc.) and executable scripts to insert these signals into resting-state EEG data. These signals can be used to decide which software or preprocessing parameters best recover the known signal while also removing artifact.

### 4.2 Reporting empirical measures of data quality

The second strategy to facilitate more robust individual difference analyses across the lifespan is the generation and evaluation of individual empirical measures of data quality with respect to denoising during pre-processing. Given that manual editing does not produce such empirical measures, until very recently as a field, we have been unable to verify that data included in analyses (almost always unavailable to reviewers or readers) were free of artifact, or whether effects of interest were influenced by artifact levels across individuals. A subset of standardized, automated software do offer such empirical quality measures following denoising (Bigdely-Shamlo et al., 2015; Debnath et al., 2020; Desjardins et al., 2021; Gabard-Durnam et al., 2018; Lopez et al., 2022; Monachino et al., 2022; Pedroni et al., 2019). However, the exact data quality measures available varies by software. We have advocated strongly for these measures’ generation and use since our first iteration of HAPPE software (Gabard-Durnam, 2018). However, EEG research is well behind other human neuroscience modalities that have shifted normative practice to include 1) reporting empirical data quality metrics in manuscripts, and 2) evaluating artifact-related measures’ impacts on brain measure variables of interest (e.g., Fishburn et al., 2019; Gratton et al., 2020; Parkes et al., 2018; Power et al., 2015; Tak and Ye, 2014). Though many EEG manuscripts report the number of artifact-free segments included in analyses, few studies report testing whether segment retention impacts their EEG measure estimates or include any information about data quality within those retained segments. For example, does the degree of retained data variance across individuals affect a feature's estimate (e.g., power in canonical frequency bands (Gabard-Durnam et al., 2018), functional connectivity values calculated with fMRI (Power et al., 2015; Pruim et al., 2015), structural MRI features (Gilmore et al., 2021))? We surely do not want to report individual differences that are driven by differences in data quality or artifact contamination instead of true neural differences. Data quality metrics may also be included in analyses as covariates (e.g., like framewise motion covariates in fMRI analyses of individual differences (Marek et al., 2019)). That is, one could include the degree of data retained in alpha frequencies after pre-processing in statistical models linking alpha power to behavior (though researchers should always evaluate potential statistical models for multicollinearity issues; see Miller and Chapman, 2001 for issues in group-difference designs with covariates that significantly differ between experimental groups).

We have far to go before there are standardized data quality measures of denoising that can regularly be evaluated and expected in EEG-based manuscripts (e.g., in the way that framewise motion data quality measures can be evaluated across MRI-based manuscripts). We offer the following suggestions to spark discussion and hopefully momentum in this direction, though. Specifically, we advocate for a minimum set of measures to be reported in methods sections and evaluated in terms of impact on EEG measure estimates reported in results: data quality measures that indicate data retention in terms of time (e.g., percent and number of retained trials), frequency (e.g., cross-correlation between data prior to denoising and following artifact denoising for a given set of frequencies or frequency band), and space (e.g., percent channels that were bad within a region of interest, or total percent bad channels for whole-head analyses). We focus on these signal properties because they offer orthogonal information about the EEG signal's quality and are relevant for the vast majority of EEG features (e.g., ERPs averaged over trials in time for a subset of frequencies in the signal, frontal alpha power calculated over specific frequencies of interest over a region of interest on the scalp). Empirical indices of data quality may therefore facilitate more rigorous testing and evaluation of individual difference effects.

### 4.3 Recommendations to improve EEG analyses for individual difference testing

We hope to see EEG researchers pivot from subjective methods of pre-processing and denoising our EEG data to more widespread adoption of standardized denoising pipelines and empirical measures of data quality to ensure robust individual difference analyses. There are currently (different) standardized, automated pipeline options that fit every type of data/analysis, though there is work to be done in determining which standardized pipelines will best serve the EEG community. To facilitate this shift in pre-processing practice, we offer the following recommendations for researchers. For those who implement new software:

1) Validate broadly across populations and ages and use or make freely available EEG datasets to facilitate comparisons with other pipelines.
2) Software should include quantitative measures of data quality and pre-processing-related changes. For those who use EEG in research: 1) Choose standardized, automated software whenever possible that is validated in your populations, ages, and EEG acquisition setup that provides empirical data quality outputs. Assess the performance of this software in your own data. 2) Report empirical measures of data and pre-processing quality for your samples in manuscript methods.
3) Analyze and report in manuscripts how the data quality measures impact EEG measures of interest and when appropriate, use quality metrics as covariates in analyses to more rigorously evaluate the robustness of individual difference results.

## 5 Conclusion

**Fig. 6:**
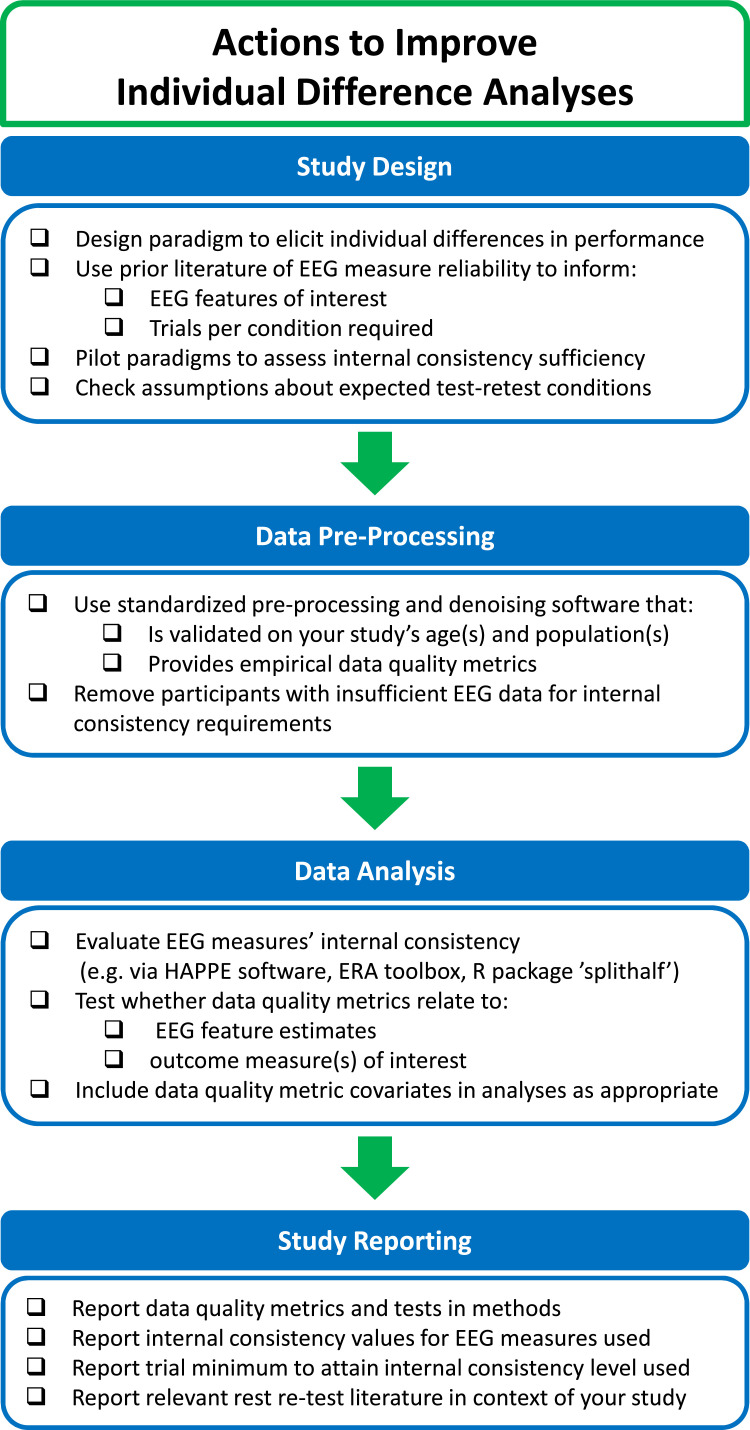
Individual actions recommended in (Lopez et al. 2023) to improve individual difference analyses with EEG data.

The potential for EEG research to inform our understanding of cognition, mental health, and behavior across the lifespan through individual differences research is greater than ever with the recent explosion of new technology, computational power, and types of extractable measures. This innovation and change also provides a key moment to reflect on how we might use this momentum to shift our scientific practices to promote robust individual differences research moving forward. Here we have focused on the role that psychometric reliability plays in EEG-based individual differences research. We have taken a developmental perspective to detail how reliability can be conceptualized across the lifespan, how it has been measured and reported across a variety of EEG-derived measures, and how several pre-processing factors may help optimize reliability across the lifespan. We see both opportunity and means for improving reliability of EEG measures at the level of individual researcher behavior. To aid individual researchers in adopting our recommendations throughout the research process, we have provided a summary checklist of actions discussed so far (Fig. 6). We can each implement these changes to make significant strides together in using EEG to understand and predict human conditions with individual difference analyses.

## Data and code availability statement

There is no data or code to share for this manuscript.

## Funding

This manuscript was supported by grant #INV-005789 from the 10.13039/100000865Bill and Melinda Gates Foundation to LGD. The authors declare no competing financial interests.

## Declaration of Competing Interest

None.

## Data Availability

No data was used for the research described in the article.

## References

[bib0001] Allen J., Urry H., Hitt S., Psychophysiology J.C. (2003). The stability of resting frontal electroencephalographic asymmetry in depression. Wiley Online Libr..

[bib0002] Allen J.J.B., Urry H.L., Hitt S.K., Coan J.A. (2004). The stability of resting frontal electroencephalographic asymmetry in depression. Psychophysiology.

[bib0003] Ambrosini E., Vallesi A. (2016). Asymmetry in prefrontal resting-state EEG spectral power underlies individual differences in phasic and sustained cognitive control. Neuroimage.

[bib0004] Anaya B., Ostlund B., LoBue V., Buss K., Pérez-Edgar K. (2021). Psychometric properties of infant electroencephalography: Developmental stability, reliability, and construct validity of frontal alpha asymmetry and delta–beta coupling. Dev. Psychobiol. Dev..

[bib0005] Andersen L.M. (2018). Group analysis in MNE-python of evoked responses from a tactile stimulation paradigm: a pipeline for reproducibility at every step of processing, going from individual sensor space representations to an across-group source space representation. Front. Neurosci..

[bib0006] Angelidis A., van der Does W., Schakel L., Putman P. (2016). Frontal EEG theta/beta ratio as an electrophysiological marker for attentional control and its test-retest reliability. Biol. Psychol..

[bib0007] Babloyantz A., Salazar J.M., Nicolis C. (1985). Evidence of chaotic dynamics of brain activity during the sleep cycle. Phys. Lett. A.

[bib0008] Bartko, J.J., 1966. The intraclass correlation coefficient as a measure of reliability. 19, 3–11. doi:10.2466/PR0.1966.19.1.3. 5942109

[bib0009] Becht A.I., Mills K.L. (2020). Modeling Individual Differences in Brain Development. Biol. Psychiatry.

[bib0010] Beker, S., Foxe, J.J., Venticinque, J., Bates, J., Ridgeway, E.M., Schaaf, R.C., Molholm, S., Org, S.M., 2021. Looking for Consistency in an Uncertain World: Test-Retest Reliability of Neurophysiological and Behavioral Readouts in Autism. doi:10.21203/RS.3.RS-240084/V1. PMC8483424 34592931

[bib0011] Bigdely-Shamlo N., Mullen T., Kothe C., Su K.-M., Robbins K.A. (2015). The PREP pipeline: standardized preprocessing for large-scale EEG analysis. Front. Neuroinform.

[bib0012] Bosl W., Tierney A., Tager-Flusberg H., Nelson C. (2011). EEG complexity as a biomarker for autism spectrum disorder risk. BMC Med..

[bib0013] Bosl W.J., Tager-Flusberg H., Nelson C.A. (2018). EEG analytics for early detection of autism spectrum disorder: a data-driven approach. Sci. Rep..

[bib0014] Botdorf, M., Rosenbaum, G.M., Patrianakos, J., Steinberg, L., Chein, J.M., 2016. Adolescent risk-taking is predicted by individual differences in cognitive control over emotional, but not non-emotional, response conflict. 31, 972–979. doi:10.1080/02699931.2016.1168285. 27050317

[bib0015] Boudewyn M.A., Luck S.J., Farrens J.L., Kappenman E.S. (2018). How many trials does it take to get a significant ERP effect? It depends. Psychophysiology.

[bib0016] Brandmaier A.M., Wenger E., Bodammer N.C., Kühn S., Raz N., Lindenberger U. (2018). Assessing reliability in neuroimaging research through intra-class effect decomposition (ICED). Elife.

[bib0017] Bresin K., Verona E. (2021). Craving and substance use: examining psychophysiological and behavioral moderators. Int. J. Psychophysiol..

[bib0018] Brunner J., Hansen T., Olsen A., Skandsen T., Håberg A., Kropotov J. (2013). Long-term test-retest reliability of the P3 NoGo wave and two independent components decomposed from the P3 NoGo wave in a visual Go/NoGo task. Int. J. Psychophysiol..

[bib0019] Bruns A., Eckhorn R., Jokeit H. (2000). Amplitude envelope correlation detects coupling among incoherent brain signals. Neuroreport.

[bib0020] Büchel D., Lehmann T., Sandbakk Ø., Baumeister J. (2021). EEG-derived brain graphs are reliable measures for exploring exercise-induced changes in brain networks. Sci. Rep..

[bib0021] Burgess A., Gruzelier J. (1993). Individual reliability of amplitude distribution in topographical mapping of EEG. Electroencephalogr. Clin. Neurophysiol..

[bib0022] Buzzell G.A., Morales S., Valadez E.A., Hunnius S., Fox N.A. (2023). Maximizing the potential of EEG as a developmental neuroscience tool. Dev. Cogn. Neurosci..

[bib0023] Cannon R., Baldwin D., Shaw T., Diloreto D., Phillips S., Scruggs A., Riehl T. (2012). Reliability of quantitative EEG (qEEG) measures and LORETA current source density at 30 days. Neurosci. Lett..

[bib0024] Carbine K.A., Clayson P.E., Baldwin S.A., LeCheminant J.D., Larson M.J. (2021). Using generalizability theory and the ERP reliability analysis (ERA) toolbox for assessing test-retest reliability of ERP scores part 2: Application to food-based tasks and stimuli. Int. J. Psychophysiol..

[bib0025] Cassani R., Falk T.H., Fraga F.J., Cecchi M., Moore D.K., Anghinah R. (2017). Towards automated electroencephalography-based Alzheimer's disease diagnosis using portable low-density devices. Biomed. Signal Process. Control.

[bib0026] Cassidy S.M., Robertson I.H., O'Connell R.G. (2012). Retest reliability of event-related potentials: Evidence from a variety of paradigms. Psychophysiology.

[bib0027] Catarino A., Andrade A., Churches O., Wagner A.P., Baron-Cohen S., Ring H. (2013). Task-related functional connectivity in autism spectrum conditions: an EEG study using wavelet transform coherence. Mol. Autism.

[bib0028] Catarino A., Churches O., Baron-Cohen S., Andrade A., Ring H. (2011). Atypical EEG complexity in autism spectrum conditions: a multiscale entropy analysis. Clin. Neurophysiol..

[bib0029] Cavanagh J.F., Kumar P., Mueller A.A., Richardson S.P., Mueen A. (2018). Diminished EEG habituation to novel events effectively classifies Parkinson's patients. Clin. Neurophysiol..

[bib0030] Chaumon M., Bishop D.V.M., Busch N.A. (2015). A practical guide to the selection of independent components of the electroencephalogram for artifact correction. J. Neurosci. Methods.

[bib0031] Chen C., Chan C.W., Cheng Y. (2018). Test–retest reliability of mismatch negativity (MMN) to emotional voices. Front. Hum. Neurosci..

[bib0032] Clarke A.D.F., Irons J.L., James W., Leber A.B., Hunt A.R. (2022). Stable individual differences in strategies within, but not between, visual search tasks. Q. J. Exp. Psychol. (Hove).

[bib0033] Clayson P., Baldwin S., Larson M. (2013). How does noise affect amplitude and latency measurement of event-related potentials (ERPs)? A methodological critique and simulation study. Psychophysiology.

[bib0034] Clayson P.E. (2020). Moderators of the internal consistency of error-related negativity scores: A meta-analysis of internal consistency estimates. Psychophysiology.

[bib0035] Clayson P.E., Brush C.J., Hajcak G. (2021). Data quality and reliability metrics for event-related potentials (ERPs): the utility of subject-level reliability. Int. J. Psychophysiol..

[bib0036] Clayson P.E., Carbine K.A., Baldwin S.A., Larson M.J. (2019). Methodological reporting behavior, sample sizes, and statistical power in studies of event-related potentials: Barriers to reproducibility and replicability. Psychophysiology.

[bib0037] Clayson P.E., Carbine K.A., Baldwin S.A., Olsen J.A., Larson M.J. (2021). Using generalizability theory and the ERP Reliability Analysis (ERA) Toolbox for assessing test-retest reliability of ERP scores part 1: Algorithms, framework, and implementation. Int. J. Psychophysiol..

[bib0038] Clayson P.E., Miller G.A. (2017). ERP Reliability Analysis (ERA) Toolbox: an open-source toolbox for analyzing the reliability of event-related brain potentials. Int. J. Psychophysiol..

[bib0039] Clayson P.E., Miller G.A. (2017). Psychometric considerations in the measurement of event-related brain potentials: guidelines for measurement and reporting. Int. J. Psychophysiol..

[bib0040] Cognitive neuroscience at the crossroads, 2022. Nature 608, 647. doi:10.1038/D41586-022-02283-W. 36002501

[bib0041] Colombo M.A., Napolitani M., Boly M., Gosseries O., Casarotto S., Rosanova M., Brichant J.F., Boveroux P., Rex S., Laureys S., Massimini M., Chieregato A., Sarasso S. (2019). The spectral exponent of the resting EEG indexes the presence of consciousness during unresponsiveness induced by propofol, xenon, and ketamine. Neuroimage.

[bib0042] Corsi-Cabrera M., Galindo-Vilchis L., del-Río-Portilla Y., Arce C., Ramos-Loyo J. (2007). Within-subject reliability and inter-session stability of EEG power and coherent activity in women evaluated monthly over nine months. Clin. Neurophysiol..

[bib0043] Cremone-Caira A., Vaidyanathan A., Hyatt D., Gilbert R., Clarkson T., Faja S. (2020). Test-retest reliability of the N2 event-related potential in school-aged children with autism spectrum disorder (ASD). Clin. Neurophysiol..

[bib0044] Cronbach L.J. (1951). Coefficient alpha and the internal structure of tests. Psychometrika.

[bib0045] da Cruz J.R., Chicherov V., Herzog M.H., Figueiredo P. (2018). An automatic pre-processing pipeline for EEG analysis (APP) based on robust statistics. Clin. Neurophysiol..

[bib0046] Dai M., Li Y., Gan S., Du F. (2019). The reliability of estimating visual working memory capacity. Sci. Rep..

[bib0047] de Aguiar Neto F.S., Rosa J.L.G. (2019). Depression biomarkers using non-invasive EEG: a review. Neurosci. Biobehav. Rev..

[bib0048] Debnath R., Buzzell G.A., Morales S., Bowers M.E., Leach S.C., Fox N.A. (2020). The Maryland analysis of developmental EEG (MADE) pipeline. Psychophysiology.

[bib0049] Demanuele C., James C.J., Sonuga-Barke E.J. (2007). Distinguishing low frequency oscillations within the 1/f spectral behaviour of electromagnetic brain signals. Behav. Brain Funct..

[bib0050] Demuru, M., Fraschini, M., 2020. EEG fingerprinting: subject specific signature based on the aperiodic component of power spectrum. 10.1016/j.compbiomed.2020.103748 32421651

[bib0051] Desjardins J.A., van Noordt S., Huberty S., Segalowitz S.J., Elsabbagh M. (2021). EEG Integrated Platform Lossless (EEG-IP-L) pre-processing pipeline for objective signal quality assessment incorporating data annotation and blind source separation. J. Neurosci. Methods.

[bib0052] Deuker L., Bullmore E.T., Smith M., Christensen S., Nathan P.J., Rockstroh B., Bassett D.S. (2009). Reproducibility of graph metrics of human brain functional networks. Neuroimage.

[bib0053] Dien J. (2010). The ERP PCA Toolkit: an open source program for advanced statistical analysis of event-related potential data. J. Neurosci. Methods.

[bib0054] Donoghue T., Haller M., Peterson E., Varma P., Sebastian P., Gao R., Noto T., Lara A., Wallis J., Knight R., Shestyuk A., Voytek B. (2020). Parameterizing neural power spectra into periodic and aperiodic components. Nat. Neurosci..

[bib0055] Drew T., Vogel E.K. (2008). Neural measures of individual differences in selecting and tracking multiple moving objects. J. Neurosci..

[bib0056] Dünki R. (2000).

[bib0057] Dushanova J.A., Tsokov S.A., Dushanova J.A., Tsokov S.A. (2020). Small-world EEG network analysis of functional connectivity in developmental dyslexia after visual training intervention. J. Integr. Neurosci..

[bib0058] Elliott M.L., Knodt A.R., Hariri A.R. (2021). Striving toward translation: strategies for reliable fMRI measurement. Trends Cogn. Sci..

[bib0059] Fallgatter A., Bartsch A., Strik W., Mueller T., Eisenack S., Neuhauser B., Aranda D., Herrmann M. (2001). Test-retest reliability of electrophysiological parameters related to cognitive motor control. Clin. Neurophysiol..

[bib0060] Fein G., Galin D., Johnstone J., Yingling C.D., Marcus M., Kiersch M.E. (1983). EEG power spectra in normal and dyslexic children. I. Reliability during passive conditions. Electroencephalogr. Clin. Neurophysiol..

[bib0061] Fernández, T., Harmony, T., Rodríguez, M., Reyes, A., Marosi, E., Bernal, J., 1993. Test-retest reliability of EEG spectral parameters during cognitive tasks: I absolute and relative power 68, 255–261. doi:10.3109/00207459308994280. 8063530

[bib0062] Figueredo A.J., Vásquez G., Brumbach B.H., Sefcek J.A., Kirsner B.R., Jacobs W.J. (2005). The K-factor: individual differences in life history strategy. Pers. Individ. Dif..

[bib0063] Fischer A.G., Klein T.A., Ullsperger M. (2017). Comparing the error-related negativity across groups: the impact of error- and trial-number differences. Psychophysiology.

[bib0064] Fishburn F.A., Ludlum R.S., Vaidya C.J., Medvedev A.V. (2019). Temporal Derivative Distribution Repair (TDDR): a motion correction method for fNIRS. Neuroimage.

[bib0065] Fisher R.A. (1958).

[bib0066] Fló A., Gennari G., Benjamin L., Dehaene-Lambertz G. (2022). Automated Pipeline for Infants Continuous EEG (APICE): a flexible pipeline for developmental cognitive studies. Dev. Cogn. Neurosci..

[bib0067] Foti, D., Kotov, R., Hajcak, G., 2013. Psychometric considerations in using error-related brain activity as a biomarker in psychotic disorders. doi:10.1037/a0032618. 23713506

[bib0068] Foulkes L., Blakemore S.J. (2018). Studying individual differences in human adolescent brain development. Nat. Neurosci..

[bib0069] Frohlich J., Reiter L.T., Saravanapandian V., DiStefano C., Huberty S., Hyde C., Chamberlain S., Bearden C.E., Golshani P., Irimia A., Olsen R.W., Hipp J.F., Jeste S.S. (2019). Mechanisms underlying the EEG biomarker in Dup15q syndrome. Mol. Autism.

[bib0070] Furman A.J., Meeker T.J., Rietschel J.C., Yoo S., Muthulingam J., Prokhorenko M., Keaser M.L., Goodman R.N., Mazaheri A., Seminowicz D.A. (2018). Cerebral peak alpha frequency predicts individual differences in pain sensitivity. Neuroimage.

[bib0071] Gabard-Durnam L.J., Mendez Leal A.S., Wilkinson C.L., Levin A.R. (2018). The Harvard automated processing pipeline for electroencephalography (HAPPE): standardized processing software for developmental and high-artifact data. Front. Neurosci..

[bib0072] Gabard-Durnam L.J., Wilkinson C., Kapur K., Tager-Flusberg H., Levin A.R., Nelson C.A. (2019). Longitudinal EEG power in the first postnatal year differentiates autism outcomes. Nat. Commun..

[bib0073] Gasser T., Bächer P., Steinberg H. (1985). Test-retest reliability of spectral parameters of the EEG. Electroencephalogr. Clin. Neurophysiol..

[bib0074] Gaudet I., Hüsser A., Vannasing P., Gallagher A. (2020). Functional brain connectivity of language functions in children revealed by EEG and MEG: a systematic review. Front. Hum. Neurosci..

[bib0075] Geuter S., Qi G., Welsh R.C., Wager T.D., Lindquist M.A. (2018). Effect size and power in fMRI group analysis. bioRxiv.

[bib0076] Gilmore A.D., Buser N.J., Hanson J.L. (2021). Variations in structural MRI quality significantly impact commonly used measures of brain anatomy. Brain Inf..

[bib0077] Gold C., Fachner J., Erkkilä J. (2013). Validity and reliability of electroencephalographic frontal alpha asymmetry and frontal midline theta as biomarkers for depression. Scand. J. Psychol..

[bib0078] Gramfort A., Luessi M., Larson E., Engemann D.A., Strohmeier D., Brodbeck C., Parkkonen L., Hämäläinen M.S. (2014). MNE software for processing MEG and EEG data. Neuroimage.

[bib0079] Gratton C., Dworetsky A., Coalson R.S., Adeyemo B., Laumann T.O., Wig G.S., Kong T.S., Gratton G., Fabiani M., Barch D.M., Tranel D., Miranda-Dominguez O., Fair D.A., Dosenbach N.U.F., Snyder A.Z., Perlmutter J.S., Petersen S.E., Campbell M.C. (2020). Removal of high frequency contamination from motion estimates in single-band fMRI saves data without biasing functional connectivity. Neuroimage.

[bib0080] Greene, A.S., Shen, X., Noble, S., Horien, C., Hahn, C.A., Arora, J., Tokoglu, F., Spann, M.N., Carrión, C.I., Barron, D.S., Sanacora, G., Srihari, V.H., Woods, S.W., Scheinost, D., Constable, R.T., 2022. Brain–phenotype models fail for individuals who defy sample stereotypes. Nat. 2022 6097925 609, 109–118. doi:10.1038/s41586-022-05118-w PMC9433326 36002572

[bib0081] Grieve P.G., Isler J.R., Izraelit A., Peterson B.S., Fifer W.P., Myers M.M., Stark R.I. (2008). EEG functional connectivity in term age extremely low birth weight infants. Clin. Neurophysiol..

[bib0082] Gudmundsson S., Runarsson T., Sigurdsson S., Eiriksdottir G., Johnsen K. (2007). Reliability of quantitative EEG features. Clin. Neurophysiol..

[bib0083] Haartsen R., Jones E.J.H., Orekhova E.V., Charman T., Johnson M.H. (2019). Functional EEG connectivity in infants associates with later restricted and repetitive behaviours in autism; a replication study. Transl. Psychiatry.

[bib0084] Haartsen R., van der Velde B., Jones E.J.H., Johnson M.H., Kemner C. (2020). Using multiple short epochs optimises the stability of infant EEG connectivity parameters. Sci. Rep..

[bib0085] Hajcak G., Meyer A., Kotov R. (2017). Psychometrics and the neuroscience of individual differences: internal consistency limits between-subjects effects. J. Abnorm. Psychol..

[bib0086] Hajra S.G., Gopinath S., Liu C.C., Pawlowski G., Fickling S.D., Song X., D'Arcy R.C.N. (2020). IEMTRONICS 2020 - International IOT, Electronics and Mechatronics Conference, Proceedings.

[bib0087] Hakim N., Awh E., Vogel E.K., Rosenberg M.D. (2021). Inter-electrode correlations measured with EEG predict individual differences in cognitive ability. Curr. Biol..

[bib0088] Hall M.H., Schulze K., Rijsdijk F., Picchioni M., Ettinger U., Bramon E., Freedman R., Murray R.M., Sham P. (2006). Heritability and reliability of P300, P50 and duration mismatch negativity. Behav. Genet..

[bib0089] Hämmerer D., Li S., Völkle M. (2012). A lifespan comparison of the reliability, test-retest stability, and signal-to-noise ratio of event-related potentials assessed during performance monitoring. Wiley Online Libr..

[bib0090] Hannesdóttir D.K., Doxie J., Bell M.A., Ollendick T.H., Wolfe C.D. (2010). A longitudinal study of emotion regulation and anxiety in middle childhood: associations with frontal EEG asymmetry in early childhood. Dev. Psychobiol..

[bib0091] Hardmeier M., Hatz F., Bousleiman H., Schindler C., Stam C.J., Fuhr P. (2014). Reproducibility of functional connectivity and graph measures based on the phase lag index (PLI) and weighted phase lag index (wPLI) derived from high resolution EEG. PLoS One.

[bib0092] Haresign, I.M., Phillips, E., Whitehorn, M., Noreika, V., Jones, E.J.H., Leong, V., Wass, S.V., 2021. Automatic classification of ICA components from infant EEG using MARA. bioRxiv 2021.01.22.427809. doi:10.1101/2021.01.22.427809. PMC8556604 34715619

[bib0093] Hatz, F., Hardmeier, M., Bousleiman, H., Rüegg, S., Schindler, C., Fuhr, P., 2016. Reliability of functional connectivity of electroencephalography applying microstate-segmented versus classical calculation of phase lag index. 6, 461–469. doi:10.1089/BRAIN.2015.0368. 27220459

[bib0094] Hatz F., Hardmeier M., Bousleiman H., Rüegg S., Schindler C., Fuhr P. (2015). Reliability of fully automated versus visually controlled pre- and post-processing of resting-state EEG. Clin. Neurophysiol..

[bib0095] He B.J., Zempel J.M., Snyder A.Z., Raichle M.E. (2010). The temporal structures and functional significance of scale-free brain activity. Neuron.

[bib0096] Hedge C., Powell G., Sumner P. (2018). The reliability paradox: why robust cognitive tasks do not produce reliable individual differences. Behav. Res. Methods.

[bib0097] Hill K., Neo W., Hernandez A. (2020).

[bib0098] Hodel A.S., Brumbaugh J.E., Hunt R.H., Van Den Heuvel S.E., Wiltgen A.M., Thomas K.M. (2019). Individual differences in ERP measures of executive function in early childhood: relation to low-risk preterm birth and parent-reported behavior. Child Neuropsychol.

[bib0099] Hounkpatin H.O., Boyce C.J., Dunn G., Wood A.M. (2018). Modeling bivariate change in individual differences: prospective associations between personality and life satisfaction. J. Pers. Soc. Psychol..

[bib0100] Hudac C.M., DesChamps T.D., Arnett A.B., Cairney B.E., Ma R., Webb S.J., Bernier R.A. (2018). Early enhanced processing and delayed habituation to deviance sounds in autism spectrum disorder. Brain Cogn..

[bib0101] Huffmeijer R., Bakermans-Kranenburg M.J., Alink L.R.A., Van IJzendoorn M.H. (2014). Reliability of event-related potentials: the influence of number of trials and electrodes. Physiol. Behav..

[bib0102] Infantolino Z.P., Luking K.R., Sauder C.L., Curtin J.J., Hajcak G. (2018). Robust is not necessarily reliable: from within-subjects fMRI contrasts to between-subjects comparisons. Neuroimage.

[bib0103] Ip C.T., Ganz M., Ozenne B., Sluth L.B., Gram M., Viardot G., l'Hostis P., Danjou P., Knudsen G.M., Christensen S.R. (2018). Pre-intervention test-retest reliability of EEG and ERP over four recording intervals. Int. J. Psychophysiol..

[bib0104] Jetha M.K., Segalowitz S.J., Gatzke-Kopp L.M. (2021). The reliability of visual ERP components in children across the first year of school. Dev. Psychobiol..

[bib0105] Jiao X., Hu Q., Tang Y., Qian Z., Tong S., Wang J., Sun J. (2022). Test-retest reliability of mismatch negativity and gamma-band auditory steady-state response in patients with schizophrenia. Schizophr. Res..

[bib0106] Jin, S.-H., Seol, J., Kim, J.S., Chung, C.K., 2011. How reliable are the functional connectivity networks of MEG in resting states? 106, 2888–2895. doi:10.1152/JN.00335.2011. 21880941

[bib0107] Jones E.J.H., Goodwin A., Orekhova E., Charman T., Dawson G., Webb S.J., Johnson M.H. (2020). Infant EEG theta modulation predicts childhood intelligence. Sci. Rep..

[bib0108] Jordan C.J., Weiss S.R.B., Howlett K.D., Freund M.P. (2020). Introduction to the special issue on “informing longitudinal studies on the effects of maternal stress and substance use on child development: planning for the HEALthy brain and child development (HBCD) study.”. Advers. Resil. Sci..

[bib0109] Kappenman E.S., Luck S.J. (2016). Best practices for event-related potential research in clinical populations. Biol. Psychiatry Cogn. Neurosci. Neuroimaging.

[bib0110] Kennedy J.T., Harms M.P., Korucuoglu O., Astafiev S.V., Barch D.M., Thompson W.K., Bjork J.M., Anokhin A.P. (2022). Reliability and stability challenges in ABCD task fMRI data. Neuroimage.

[bib0111] Keune P.M., Hansen S., Sauder T., Jaruszowic S., Kehm C., Keune J., Weber E., Schönenberg M., Oschmann P. (2019). Frontal brain activity and cognitive processing speed in multiple sclerosis: an exploration of EEG neurofeedback training. NeuroImage Clin..

[bib0112] Kinoshita S., Inoue M., Maeda H., Nakamura J., Morita K. (1996). Long-term patterns of change in ERPs across repeated measurements. Physiol. Behav..

[bib0113] Knyazev G.G., Savostyanov A.N., Bocharov A.V., Tamozhnikov S.S., Kozlova E.A., Leto I.V., Slobodskaya H.R. (2019). Cross-frequency coupling in developmental perspective. Front. Hum. Neurosci..

[bib0114] Koller-Schlaud K., Querbach J., Behr J., Ströhle A., Rentzsch J. (2020). Test-retest reliability of frontal and parietal alpha asymmetry during presentation of emotional face stimuli in healthy subjects. Neuropsychobiology.

[bib0115] Kompatsiari K., Candrian G., Mueller A. (2016). Test-retest reliability of ERP components: a short-term replication of a visual Go/NoGo task in ADHD subjects. Neurosci. Lett..

[bib0116] Kragel, P.A., Han, X., Kraynak, T.E., Gianaros, P.J., Wager, T.D., 2021. Functional MRI can be highly reliable, but it depends on what you measure: a commentary on Elliott et al. (2020). Psychol. Sci. 32, 622–626. doi:10.1177/0956797621989730/ASSET/IMAGES/LARGE/10.1177_0956797621989730-FIG1.JPEG. PMC8258303 33685310

[bib0117] Kujawa A., Carroll A., Mumper E., Mukherjee D., Kessel E., Olino T., Hajcak G., Klein D. (2018). A longitudinal examination of event-related potentials sensitive to monetary reward and loss feedback from late childhood to middle adolescence. Int. J. Psychophysiol..

[bib0118] Kujawa A., Klein D.N., Proudfit G.H. (2013). Two-year stability of the late positive potential across middle childhood and adolescence. Biol. Psychol..

[bib0119] Kuntzelman K., Jack Rhodes L., Harrington L.N., Miskovic V. (2018). A practical comparison of algorithms for the measurement of multiscale entropy in neural time series data. Brain Cogn..

[bib0120] Kuntzelman K., Miskovic V. (2017). Reliability of graph metrics derived from resting-state human EEG. Psychophysiology.

[bib0121] Larson M.J., Baldwin S.A., Good D.A., Fair J.E. (2010). Brief Reports: temporal stability of the error-related negativity (ERN) and post-error positivity (Pe): the role of number of trials. Psychophysiology.

[bib0122] Lawhern V., Hairston W.D., Robbins K. (2013). DETECT: a MATLAB toolbox for event detection and identification in time series, with applications to artifact detection in EEG signals. PLoS One.

[bib0123] Leach S.C., Morales S., Bowers M.E., Buzzell G.A., Debnath R., Beall D., Fox N.A. (2020). Adjusting ADJUST: optimizing the ADJUST algorithm for pediatric data using geodesic nets. Psychophysiology.

[bib0124] Levin, A.R., Gabard-Durnam, L.J., Mendez Leal, A.S., O'Leary, H.M., Wilkinson, C.L., Tager-Flusberg, H., Nelson, C.A., 2017. Infant sibling project: sample files. doi:10.5281/ZENODO.998965.

[bib0125] Levin A.R., Naples A.J., Scheffler A.W., Webb S.J., Shic F., Sugar C.A., Murias M., Bernier R.A., Chawarska K., Dawson G., Faja S., Jeste S., Nelson C.A., McPartland J.C., Şentürk D. (2020). Day-to-day test-retest reliability of EEG profiles in children with autism spectrum disorder and typical development. Front. Integr. Neurosci..

[bib0126] Levinson A., Speed B., Infantolino Z., Hajcak G. (2017). Reliability of the electrocortical response to gains and losses in the doors task. Psychophysiology.

[bib0127] Lew H.L., Gray M., Poole J.H. (2007). Temporal stability of auditory event-related potentials in healthy individuals and patients with traumatic brain injury. J. Clin. Neurophysiol..

[bib0128] Light G.A., Braff D.L. (2005). Stability of mismatch negativity deficits and their relationship to functional impairments in chronic schizophrenia. Am. J. Psychiatry.

[bib0129] Lin M., Davies P., Stephens J., Gavin W. (2020). Test-retest reliability of electroencephalographic measures of performance monitoring in children and adults. Dev. Neuropsychol..

[bib0130] Lopez K.L., Monachino A.D., Morales S., Leach S.C., Bowers M.E., Gabard-Durnam L.J. (2022). HAPPILEE: HAPPE In Low Electrode Electroencephalography, a standardized pre-processing software for lower density recordings. Neuroimage.

[bib0131] Lopez, K.L., Monachino, A.D., Morales, S., Leach, S.C., Bowers, M.E., Gabard-Durnam, L.J., 2021. HAPPILEE: The Harvard Automated Processing Pipeline In Low Electrode Electroencephalography, a standardized software for low density EEG and ERP data. bioRxiv 2021.07.02.450940. doi:10.1101/2021.07.02.450940.

[bib0132] Luck S.J., Gaspelin N. (2017). How to get statistically significant effects in any ERP experiment (and why you shouldn't). Psychophysiology.

[bib0133] Luck S.J., Stewart A.X., Simmons A.M., Rhemtulla M. (2021). Standardized measurement error: a universal metric of data quality for averaged event-related potentials. Psychophysiology.

[bib0134] Luking K.R., Nelson B.D., Infantolino Z.P., Sauder C.L., Hajcak G. (2017). Internal consistency of functional magnetic resonance imaging and electroencephalography measures of reward in late childhood and early adolescence. Biol. Psychiatry Cogn. Neurosci. Neuroimaging.

[bib0135] Lund T.R., Sponheim S.R., Iacono W.G., Clementz B.A. (1995). Internal consistency reliability of resting EEG power spectra in schizophrenic and normal subjects. Psychophysiology.

[bib0136] MacDonald J.A., Trafimow D. (2013). A measure of within-participant response consistency. Behav. Res. Methods.

[bib0137] MacDonald S.W.S., Nyberg L., Bäckman L. (2006). Intra-individual variability in behavior: links to brain structure, neurotransmission and neuronal activity. Trends Neurosci..

[bib0138] Malcolm B.R., Foxe J.J., Butler J.S., Mowrey W.B., Molholm S., Sanctis P.De (2019). Long-term test-retest reliability of event-related potential (ERP) recordings during treadmill walking using the mobile brain/body imaging (MoBI) approach. Brain Res..

[bib0139] Marek S., Tervo-Clemmens B., Nielsen A.N., Wheelock M.D., Miller R.L., Laumann T.O., Earl E., Foran W.W., Cordova M., Doyle O., Perrone A., Miranda-Dominguez O., Feczko E., Sturgeon D., Graham A., Hermosillo R., Snider K., Galassi A., Nagel B.J., Ewing S.W.F., Eggebrecht A.T., Garavan H., Dale A.M., Greene D.J., Barch D.M., Fair D.A., Luna B., Dosenbach N.U.F. (2019). Identifying reproducible individual differences in childhood functional brain networks: An ABCD study. Dev. Cogn. Neurosci..

[bib0140] Marshall P.J., Bar-Haim Y., Fox N.A. (2002). Development of the EEG from 5 months to 4 years of age. Clin. Neurophysiol..

[bib0141] Mathalon D.H., Sohal V.S. (2015). Neural oscillations and synchrony in brain dysfunction and neuropsychiatric disorders: it's about time. JAMA Psychiatry.

[bib0142] McDonnell M.D., Ward L.M. (2011). The benefits of noise in neural systems: bridging theory and experiment. Nat. Rev. Neurosci..

[bib0143] McEvoy L.K., Smith M.E., Gevins A. (2000). Test–retest reliability of cognitive EEG. Clin. Neurophysiol..

[bib0144] McPartland J.C., Bernier R.A., Jeste S.S., Dawson G., Nelson C.A., Chawarska K., Earl R., Faja S., Johnson S.P., Sikich L., Brandt C.A., Dziura J.D., Rozenblit L., Hellemann G., Levin A.R., Murias M., Naples A.J., Platt M.L., Sabatos-DeVito M., Shic F., Senturk D., Sugar C.A., Webb S.J. (2020). The autism biomarkers consortium for clinical trials (ABC-CT): scientific context, study design, and progress toward biomarker qualification. Front. Integr. Neurosci..

[bib0145] Metzen D., Genc E., Getzmann S., Larra M., Wascher E., Ocklenburg S. (2021). Frontal and parietal EEG alpha asymmetry: a large-scale investigation of short-term reliability on distinct EEG systems. Res. Sq..

[bib0146] Meyer A., Bress J., Hajcak Proudfit G. (2014). Psychometric properties of the error-related negativity in children and adolescents. Psychophysiology.

[bib0147] Meyer A., Riesel A., Hajcak Proudfit G. (2013). Reliability of the ERN across multiple tasks as a function of increasing errors. Psychophysiology.

[bib0148] Miller G.A., Chapman J.P. (2001). Misunderstanding analysis of covariance. J. Abnorm. Psychol..

[bib0149] Miskovic V., Keil A. (2015). Reliability of event-related EEG functional connectivity during visual entrainment: magnitude squared coherence and phase synchrony estimates. Psychophysiology.

[bib0150] Mognon A., Jovicich J., Bruzzone L., Buiatti M. (2011). ADJUST: an automatic EEG artifact detector based on the joint use of spatial and temporal features. Psychophysiology.

[bib0151] Monachino A.D., Lopez K.L., Pierce L.J., Gabard-Durnam L.J. (2022). The HAPPE plus Event-Related (HAPPE+ER) software: a standardized preprocessing pipeline for event-related potential analyses. Dev. Cogn. Neurosci..

[bib0152] Monachino, A.D., Lopez, K.L., Pierce, L.J., Gabard-Durnam, L.J., 2021. The HAPPE plus Event-Related (HAPPE+ER) software: a standardized processing pipeline for event-related potential analyses. bioRxiv 2021.07.02.450946. doi:10.1101/2021.07.02.450946. PMC9356149 35926469

[bib0153] Morales S., Bowers M.E., Leach S.C., Buzzell G.A., Fifer W., Elliott A.J., Fox N.A. (2022). Time–frequency dynamics of error monitoring in childhood: an EEG study. Dev. Psychobiol..

[bib0154] Moser, J.S., Durbin, C.E., Patrick, C.J., Schmidt, N.B., 2015. Combining neural and behavioral indicators in the assessment of internalizing psychopathology in children and adolescents. 44, 329–340. doi:10.1080/15374416.2013.865191. 24484405

[bib0155] Mullen T., Kothe C., Chi Y.M., Ojeda A., Kerth T., Makeig S., Cauwenberghs G., Jung T.P. (2013). Proceedings of the Annual International Conference of the IEEE Engineering in Medicine and Biology Society.

[bib0156] Munsters N.M., van Ravenswaaij H., van den Boomen C., Kemner C. (2019). Test-retest reliability of infant event related potentials evoked by faces. Neuropsychologia.

[bib0157] Namazi, H., Jafari, S., 2019. Estimating of brain development in newborns by fractal analysis of sleep electroencephalographic (EEG) signal. doi:10.1142/S0218348x1950021X.

[bib0158] Näpflin M., Wildi M., Sarnthein J. (2008). Test–retest reliability of EEG spectra during a working memory task. Neuroimage.

[bib0159] Näpflin M., Wildi M., Sarnthein J. (2007). Test–retest reliability of resting EEG spectra validates a statistical signature of persons. Clin. Neurophysiol..

[bib0160] Noble S., Scheinost D., Constable R.T. (2021). A guide to the measurement and interpretation of fMRI test-retest reliability. Curr. Opin. Behav. Sci..

[bib0161] Nolan H., Whelan R., Reilly R.B. (2010). FASTER: fully automated statistical thresholding for EEG artifact rejection. J. Neurosci. Methods.

[bib0162] O'Reilly C., Lewis J.D., Elsabbagh M. (2017). Is functional brain connectivity atypical in autism? A systematic review of EEG and MEG studies. PLoS One.

[bib0163] Olvet D.M., Hajcak G. (2009). Reliability of error-related brain activity. Brain Res..

[bib0164] Olvet D.M., Hajcak G. (2009). The stability of error-related brain activity with increasing trials. Psychophysiology.

[bib0165] Oostenveld R., Fries P., Maris E., Schoffelen J.M. (2011). FieldTrip: open source software for advanced analysis of MEG, EEG, and invasive electrophysiological data. Comput. Intell. Neurosci..

[bib0166] Ostlund B., Donoghue T., Anaya B., Gunther K.E., Karalunas S.L., Voytek B., Pérez-Edgar K.E. (2022). Spectral parameterization for studying neurodevelopment: how and why. Dev. Cogn. Neurosci..

[bib0167] Ostlund B.D., Alperin B.R., Drew T., Karalunas S.L. (2021). Behavioral and cognitive correlates of the aperiodic (1/f-like) exponent of the EEG power spectrum in adolescents with and without ADHD. Dev. Cogn. Neurosci..

[bib0168] Ouyang G., Dien J., Lorenz R. (2022). Handling EEG artifacts and searching individually optimal experimental parameter in real time: a system development and demonstration. J. Neural Eng..

[bib0169] Ozonoff S., Young G.S., Carter A., Messinger D., Yirmiya N., Zwaigenbaum L., Bryson S., Carver L.J., Constantino J.N., Dobkins K., Hutman T., Iverson J.M., Landa R., Rogers S.J., Sigman M., Stone W.L. (2011). Recurrence risk for autism spectrum disorders: a baby siblings research consortium study. Pediatrics.

[bib0170] Parkes L., Fulcher B., Yücel M., Fornito A. (2018). An evaluation of the efficacy, reliability, and sensitivity of motion correction strategies for resting-state functional MRI. Neuroimage.

[bib0171] Parsons S. (2021). splithalf: robust estimates of split half reliability. J. Open Source Softw..

[bib0172] Parsons, S., Kruijt, A.-W., Fox, E., 2019. Psychological science needs a standard practice of reporting the reliability of cognitive-behavioral measurements: 2, 378–395. doi:10.1177/2515245919879695.

[bib0173] Pat N., Wang Y., Bartonicek A., Candia J., Stringaris A. (2022). Explainable machine learning approach to predict and explain the relationship between task-based fMRI and individual differences in cognition. Cereb. Cortex..

[bib0174] Pathania A., Schreiber M., Miller M.W., Euler M.J., Lohse K.R. (2021). Exploring the reliability and sensitivity of the EEG power spectrum as a biomarker. Int. J. Psychophysiol..

[bib0175] Peck F.C., Gabard-Durnam L.J., Wilkinson C.L., Bosl W., Tager-Flusberg H., Nelson C.A. (2021). Prediction of autism spectrum disorder diagnosis using nonlinear measures of language-related EEG at 6 and 12 months. J. Neurodev. Disord..

[bib0176] Pedroni A., Bahreini A., Langer N. (2019). Automagic: standardized preprocessing of big EEG data. Neuroimage.

[bib0177] Podvalny, E., Noy, N., Harel, M., Bickel, S., Chechik, G., Schroeder, C.E., Mehta, A.D., Tsodyks, M., Malach, R., 2015. A unifying principle underlying the extracellular field potential spectral responses in the human cortex. 114, 505–519. doi:10.1152/JN.00943.2014. PMC4509389 25855698

[bib0178] Põld T., Päeske L., Hinrikus H., Lass J., Bachmann M. (2021). Long-term stability of resting state EEG-based linear and nonlinear measures. Int. J. Psychophysiol..

[bib0179] Pollock V.E., Schneider L.S., Lyness S.A. (1991). Reliability of topographic quantitative EEG amplitude in healthy late-middle-aged and elderly subjects. Electroencephalogr. Clin. Neurophysiol..

[bib0180] Pontifex M.B., Scudder M.R., Brown M.L., O'Leary K.C., Wu C.T., Themanson J.R., Hillman C.H. (2010). On the number of trials necessary for stabilization of error-related brain activity across the life span. Psychophysiology.

[bib0181] Power J.D., Schlaggar B.L., Petersen S.E. (2015). Recent progress and outstanding issues in motion correction in resting state fMRI. Neuroimage.

[bib0182] Pruim R.H.R., Mennes M., van Rooij D., Llera A., Buitelaar J.K., Beckmann C.F. (2015). ICA-AROMA: a robust ICA-based strategy for removing motion artifacts from fMRI data. Neuroimage.

[bib0183] Puglia M.H., Krol K.M., Missana M., Williams C.L., Lillard T.S., Morris J.P., Connelly J.J., Grossmann T. (2020). Epigenetic tuning of brain signal entropy in emergent human social behavior. BMC Med..

[bib0184] Puglia, M.H., Slobin, J.S., Williams, C.L., 2021. The Automated Preprocessing Pipe-Line for the Estimation of Scale-wise Entropy from EEG Data (APPLESEED): Development and validation for use in pediatric populations. bioRxiv 2021.07.10.450198. doi:10.1101/2021.07.10.450198. PMC9586850 36270100

[bib0185] Qin S., Young C.B., Duan X., Chen T., Supekar K., Menon V. (2014). Amygdala subregional structure and intrinsic functional connectivity predicts individual differences in anxiety during early childhood. Biol. Psychiatry.

[bib0186] Rentzsch J., Jockers-Scherübl M., Boutros N., Gallinat J. (2008). Test-retest reliability of P50, N100 and P200 auditory sensory gating in healthy subjects. Int. J. Psychophysiol..

[bib0187] Righi G., Tierney A.L., Tager-Flusberg H., Nelson C.A. (2014). Functional connectivity in the first year of life in infants at risk for autism spectrum disorder: an EEG study. PLoS One.

[bib0188] Rocha H.A., Marks J., Woods A.J., Staud R., Sibille K., Keil A. (2020). *Re*-test reliability and internal consistency of EEG alpha-band oscillations in older adults with chronic knee pain. Clin. Neurophysiol..

[bib0189] Rodrigues J., Weiß M., Hewig J., Allen J.J.B. (2021). EPOS: EEG Processing Open-Source Scripts. Front. Neurosci..

[bib0190] Salinsky M.C., Oken B.S., Morehead L. (1991). Test-retest reliability in EEG frequency analysis. Electroencephalogr. Clin. Neurophysiol..

[bib0191] Sanchez-Alonso S., Aslin R.N. (2020). Predictive modeling of neurobehavioral state and trait variation across development. Dev. Cogn. Neurosci..

[bib0192] Sandre A., Banica I., Riesel A., Flake J., Klawohn J., Weinberg A. (2020). Comparing the effects of different methodological decisions on the error-related negativity and its association with behaviour and gender. Int. J. Psychophysiol..

[bib0193] Sargolzaei S., Cabrerizo M., Sargolzaei A., Noei S., Eddin A.S., Rajaei H., Pinzon-Ardila A., Gonzalez-Arias S.M., Jayakar P., Adjouadi M. (2015). A probabilistic approach for pediatric epilepsy diagnosis using brain functional connectivity networks. BMC Bioinforma..

[bib0194] Schmidt L.A., Santesso D.L., Miskovic V., Mathewson K.J., McCabe R.E., Antony M.M., Moscovitch D.A. (2012). Test–retest reliability of regional electroencephalogram (EEG) and cardiovascular measures in social anxiety disorder (SAD). Int. J. Psychophysiol..

[bib0195] Segalowitz S.J., Santesso D.L., Murphy T.I., Homan D., Chantziantoniou D.K., Khan S. (2010). Retest reliability of medial frontal negativities during performance monitoring. Psychophysiology.

[bib0196] Sinha F., Bernardy N., Parsons O.A. (1992). Long-term test-retest reliability of event-related potentials in normals and alcoholics. Biol. Psychiatry.

[bib0197] Sorella S., Vellani V., Siugzdaite R., Feraco P., Grecucci A. (2022). Structural and functional brain networks of individual differences in trait anger and anger control: An unsupervised machine learning study. Eur. J. Neurosci..

[bib0198] Soveri A., Lehtonen M., Karlsson L.C., Lukasik K., Antfolk J., Laine M. (2018). Test–retest reliability of five frequently used executive tasks in healthy adults. Appl. Neuropsychol..

[bib0199] Stam C.J. (2005). Nonlinear dynamical analysis of EEG and MEG: review of an emerging field. Clin. Neurophysiol..

[bib0200] Stewart J.L., Coan J.A., Towers D.N., Allen J.J.B. (2011). Frontal EEG asymmetry during emotional challenge differentiates individuals with and without lifetime major depressive disorder. J. Affect. Disord..

[bib0201] Streiner, D.L., 2010. Starting at the beginning: an introduction to coefficient alpha and internal consistency. 80, 99–103. doi:10.1207/S15327752JPA8001_18. 12584072

[bib0202] Strube M.J., Newman L.C., Cacioppo J.C., Tassinary L.G., Berntson G.G. (2007). The Handbook of Psychophysiology.

[bib0203] Suárez-Revelo J.X., Ochoa-Gómez J.F., Duque-Grajales J., Montoya-Betancur A., Sánchez-López S. (2015). 2015 20th Symp. Signal Process. Images Comput. Vision, STSIVA 2015 - Conf. Proc.

[bib0204] Suchan F., Kopf J., Althen H., Reif A., Plichta M.M. (2019). Reliable and efficient recording of the error-related negativity with a speeded Eriksen Flanker Task. Acta Neuropsychiatr..

[bib0205] Tadel F., Baillet S., Mosher J., Pantazis D., Leahy R. (2011). Brainstorm: a user-friendly application for MEG/EEG analysis. Comput. Intell. Neurosci..

[bib0206] Tak S., Ye J.C. (2014). Statistical analysis of fNIRS data: A comprehensive review. Neuroimage.

[bib0207] Tang W., Cui Y., Babenko O. (2014). Internal consistency: do we really know what it is and how to assess it?. J. Psychol. Behav. Sci..

[bib0208] Taylor B.K., Gavin W.J., Davies P.L. (2016). The test-retest reliability of the visually-evoked contingent negative variation (CNV) in children and adults. Dev. Neuropsychol..

[bib0209] Tenke C.E., Kayser J., Alvarenga J.E., Abraham K.S., Warner V., Talati A., Weissman M.M., Bruder G.E. (2018). Temporal stability of posterior EEG alpha over twelve years. Clin. Neurophysiol..

[bib0210] Thesen T., Murphy C. (2002). Reliability analysis of event-related brain potentials to olfactory stimuli. Psychophysiology.

[bib0211] Thigpen N.N., Kappenman E.S., Keil A. (2017). Assessing the internal consistency of the event-related potential: an example analysis. Psychophysiology.

[bib0212] Thompson B. (2003).

[bib0213] Tomarken A.J., Davidson R.J., Wheeler R.E., Kinney L. (1992). Psychometric properties of resting anterior EEG asymmetry: temporal stability and internal consistency. Psychophysiology.

[bib0214] Towers D., Psychophysiology J.A. (2008). A better estimate of the internal consistency reliability of frontal EEG asymmetry scores. Wiley Online Libr..

[bib0215] Trafimow D., Rice S. (2009). Potential performance theory (PPT): describing a methodology for analyzing task performance. Behav. Res. Methods.

[bib0216] Trenado C., González-Ramírez A., Lizárraga-Cortés V., Leal N.P., Manjarrez E., Ruge D. (2018). The potential of trial-by-trial variabilities of ongoing-EEG, evoked potentials, event related potentials and fMRI as diagnostic markers for neuropsychiatric disorders. Front. Neurosci..

[bib0217] Troller-Renfree S.V., Morales S., Leach S.C., Bowers M.E., Debnath R., Fifer W.P., Fox N.A., Noble K.G. (2021). Feasibility of assessing brain activity using mobile, in-home collection of electroencephalography: methods and analysis. Dev. Psychobiol..

[bib0218] Vacha-Haase, T., 2016. Reliability generalization: exploring variance in measurement error affecting score reliability across studies. 58, 6–20. doi:10.1177/0013164498058001002.

[bib0219] van Noordt S., Willoughby T. (2021). Cortical maturation from childhood to adolescence is reflected in resting state EEG signal complexity. Dev. Cogn. Neurosci..

[bib0220] Velde B.V.D., Haartsen R., Kemner C. (2019). Test-retest reliability of EEG network characteristics in infants. Brain Behav..

[bib0221] Vincent K., Xie W., Psychobiology C.N.-D. (2021).

[bib0222] Vogel E.K., Machizawa M.G. (2004). Neural activity predicts individual differences in visual working memory capacity. Nat..

[bib0223] Vogel E.K., McCollough A.W., Machizawa M.G. (2005). Neural measures reveal individual differences in controlling access to working memory. Nat..

[bib0224] Walhovd K., Fjell A. (2002). One-year test-retest reliability of auditory ERPs in young and old adults. Int. J. Psychophysiol..

[bib0225] Waltmann M., Schlagenhauf F., Deserno L. (2022). Sufficient reliability of the behavioral and computational readouts of a probabilistic reversal learning task. Behav. Res. Methods.

[bib0226] Wang J., Chen T., Jiao X., Liu K., Tong S., Sun J. (2021). Test-retest reliability of duration-related and frequency-related mismatch negativity. Neurophysiol. Clin..

[bib0227] Webb, S.J., Naples, A.J., Levin, A.R., Hellemann, G., Borland, H., Benton, J., Carlos, C., McAllister, T., Santhosh, M., Seow, H., Atyabi, A., Bernier, R., Chawarska, K., Dawson, G., Dziura, J., Faja, S., Jeste, S., Murias, M., Nelson, C.A., Sabatos-DeVito, M., Senturk, D., Shic, F., Sugar, C.A., McPartland, J.C., 2022. The autism biomarkers consortium for clinical trials: initial evaluation of a battery of candidate EEG biomarkers. doi:10.1176/appi.ajp.21050485. PMC10027395 36000217

[bib0228] Weinberg A., Hajcak G. (2011). Longer term test-retest reliability of error-related brain activity. Psychophysiology.

[bib0229] Wen H., Liu Z. (2016). Separating fractal and oscillatory components in the power spectrum of neurophysiological signal. Brain Topogr..

[bib0230] Wilkinson C.L., Gabard-Durnam L.J., Kapur K., Tager-Flusberg H., Levin A.R., Nelson C.A. (2020). Use of longitudinal EEG measures in estimating language development in infants with and without familial risk for autism spectrum disorder. Neurobiol. Lang..

[bib0231] Winegust, A., … K.M.-I.J. of, 2014, undefined, 2014. Test–retest reliability of frontal alpha electroencephalogram (EEG) and electrocardiogram (ECG) measures in adolescents: a pilot study. Taylor Fr. 124, 908–911. doi:10.3109/00207454.2014.895003. 24617292

[bib0232] Winkler I., Brandl S., Horn F., Waldburger E., Allefeld C., Tangermann M. (2014). Robust artifactual independent component classification for BCI practitioners. J. Neural Eng..

[bib0233] Winkler I., Haufe S., Tangermann M. (2011). Automatic classification of artifactual ICA-components for artifact removal in EEG signals. Behav. Brain Funct..

[bib0234] Xie W., Jensen S.K.G., Wade M., Kumar S., Westerlund A., Kakon S.H., Haque R., Petri W.A., Nelson C.A. (2019). Growth faltering is associated with altered brain functional connectivity and cognitive outcomes in urban Bangladeshi children exposed to early adversity. BMC Med..

[bib0235] Xu X., Inzlicht M. (2015). Neurophysiological responses to gun-shooting errors. Int. J. Psychophysiol..

[bib0236] Yen M., Lo L.-H. (2002). Examining test-retest reliability: an intra-class correlation. Nurs. Res..

[bib0237] Zhang D., Ding H., Liu Y., Zhou C., Ding H., Ye D. (2009). Neurodevelopment in newborns: a sample entropy analysis of electroencephalogram. Physiol. Meas..

